# Pluronic-F127/Platelet Microvesicles nanocomplex delivers stem cells in high doses to the bone marrow and confers post-irradiation survival

**DOI:** 10.1038/s41598-019-57057-8

**Published:** 2020-01-13

**Authors:** Vikas Chander, Gurudutta Gangenahalli

**Affiliations:** Division of Stem Cell and Gene Therapy Research, Institute of Nuclear Medicine and Allied Sciences, Defence Research and Development Organization, Delhi, 110054 India

**Keywords:** Haematopoietic stem cells, Stem-cell research

## Abstract

Platelet microvesicles (pMVs) are submicron-sized heterogeneous vesicles released upon activation and contain several membrane receptors and proteins (CD41, CD61, CD62, CXCR4, PAR-1, etc.). We have revealed their ability to adhere to the triblock copolymer pluronic-F127 (PF127) and form a platelet microvesicular nanocloud which has the potential to enhance the transvascular migration of hematopoietic stem cells across the sinusoidal endothelium to the bone marrow. Besides, the pMVs nanoclouds bestow survival benefits when present on the cells used for infusion, particularly with PF127-stabilized with chitosan-alginate (PF127-CA HSCs). The vesicles were found to be firmly associated with PF127 in the nanocloud, which was detected by confocal laser scanning microscopy. The abrogation of CXCR4/SDF-1 axis regulating the transmigration of the cells by antagonist AMD3100 revealed that the enriched CXCR4 receptors on pMVs robustize the transmigration of the infused cells. The homing of the cells led to effective engraftment and faster regeneration of the critical blood lineages, which elicited 100% survival of the mice receiving lethal doses of radiation. The Human Long-Term Culture Initiating Cells (LTC-ICs), Severe Combined Immunodeficient (SCID) - Repopulating Cells (SRCs) and Colony Forming Cells (CFCs) responsible for the regeneration, but present in extremely low numbers in the infused cell dose, have enabled the cells to reach the bone marrow in high numbers. This potential of the PF127 to sequester the pMVs and its application to achieve over 10-fold delivery of HSCs across the trans-endothelial checkpoint has so far not been reported. Thus, this mechanistic innovation is a potential post-exposure life-saving regimen capable of circumventing the irreparable damage to the bone marrow caused by lethal doses of radiation.

## Introduction

Pluronics, also known as poloxamers, are amphiphilic triblock copolymers consisting of hydrophobic polyoxypropylene (PPO) and hydrophilic polyoxyethylene (PEO) domains. Owing to their physiological properties of low toxicity and high biocompatibility, these materials are used in many pharmaceutical applications that require solubilization or stabilization of the compounds. Their rich phase behavior (micelles, hydrogels, self-assembling and repelling behaviors, etc.) makes them amenable to multiple processes and product forms^1,2^. In the latter application, their repelling behavior is exploited to minimize or abolish the fatal thrombogenic cascade, which occurs when the platelets are activated by extending their hydrophilic PEO domains, thereby reducing protein adsorption and platelet aggregation on the surface^3,4^. These properties are invaluable in different pathophysiological conditions such as leukemia, septicemia, nephritic syndromes, thrombophilia, and cardiovascular diseases. These polymers are also utilized in DNA delivery technologies, brain injury management, ophthalmic treatment, and drug delivery systems^5–7^.

The mechanistic basis and mode of interaction (both attraction and repulsion) are critical for the potential applications of these copolymers. These properties are strictly dependent on factors such as molecular weight (MW), ratio of PEO to PPO, mass ratio of PEO to protein, and Vander Waal attraction between the protein and PEO chains. Contemporary data suggest that the longer PEO chains are more adept at interacting with proteins in aqueous solutions than the shorter ones. The increased hydrophobic cavities provide multiple protein binding sites and facilitate the formation of PEO-protein complexes via hydrogen bonding with positively charged amino acids^8–10^. Optimum PEO-PPO ratio is the decisive factor which not only controls the MW of the copolymer but also the chain length and hence the degree to which the interaction takes place. Ahmed *et al*.^4^ showed that <10% coverage of the platelet surface by these pluronics is sufficient to prevent aggregation and thrombosis, which implies that certain molecules associated with the cells play a critical role in the mechanism of pluronics by directly interacting with the platelets. It was also observed that pluronics not only bind to platelets in a dose-dependent manner but also adhere to the soluble components in blood plasma^3,4,11^.

**Figure 1:**
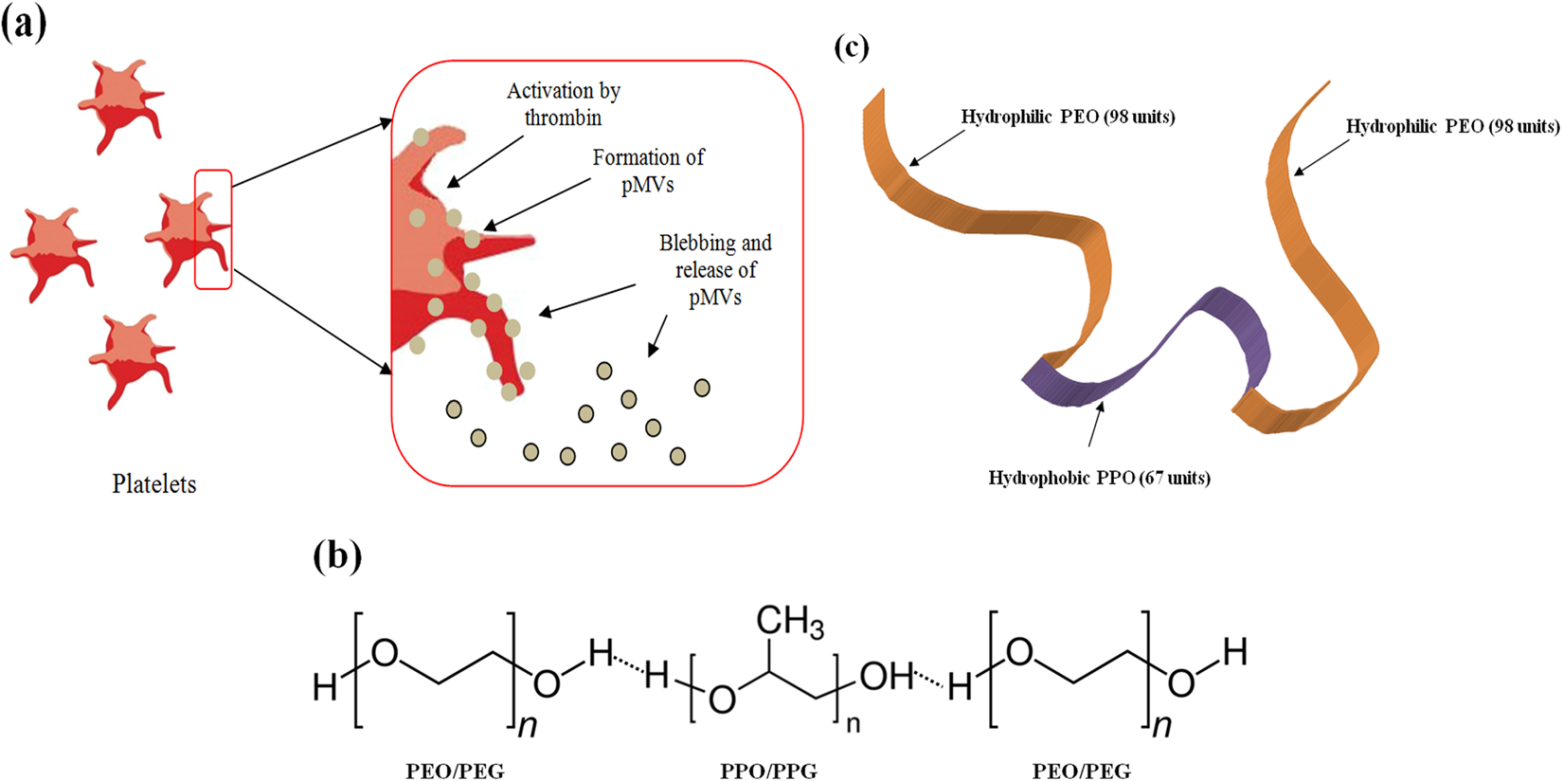
(**a**) Diagram representing the formation of pMVs. (**b**,**c**) **Structure of PF127**: PF127 consists of 67 units of hydrophobic Polyoxypropylene oxide (PPO) flanked by 98 units of hydrophilic polyethylene oxide (PEO) from both ends.

One of the most abundant plasma components is the platelet microvesicles (pMVs). These are the antecedents of the platelets and are formed from the activated or apoptotic platelets through shedding^12^ (Fig. 1(a)). The pMVs retain several platelet-associated molecules such as GPIIb/IIIa complex, integrins, CXCR4, lysophosphatidyl choline, etc., and also the platelet properties to some extent^13^. The mechanistic behavior of ensemble pMVs in the form of natural aggregates is similar to that of platelets and regulates different localized mechanisms, particularly across the vascular endothelium, which induces certain pathophysiological responses such as homeostasis, thrombosis, atherosclerosis, and inflammation^14–17^. When these pMVs are anchored to CXCR4 and other rolling and adhesion integrins, they tend to be responsive to SDF1 (CXCL12), a gradient (CXCR4/CXCL12 axis) that is consistently present only across the bone marrow due to secretions from the sinusoidal endothelium. This secretion enables them to adhere and roll, finally leading to transvascular migration (TVM). The above phenomenon has already been established to depend on the interaction between the similar molecules that are present on the surface of the infused cells and the sinusoidal endothelium^18^.

The plasma extracellular vesicles (EVs), of which the pMVs are also a major component, were particularly found to bind with pluronics. In their study, Zhong *et al*. were able to selectively enrich the EVs from the plasma by using Pluronic-F68 (PF68)^19^. We through our study deduced that the pMVs were able to bind Pluronic-F127 (PF127 or poloxamer407) more efficiently than PF68. The former consists of 67 units of PPO flanked by 98 units of PEO on both ends (Fig. 1(b,c)), possesses an average MW of 12.7 kDa, and contains 84.6 mol% PEO. Thus, we explored the likelihood of ensemble pMVs when densely made to present as platelet dust (also known as nanocloud) around the haematopoietic stem/progenitor cells (HSCs/HSPCs). We achieved this by grafting the triblock copolymer PF127 on a chitosan-alginate stabilized template over the cell surface to mediate the TVM of infused HSCs into the bone marrow. For the first time, we report the potential of this copolymer to capture the pMVs, in line with the differentially regulated ability, owing to the PEO/PPO ratios among their series. We, for the first time, also report the potential application of such platelet dust cluster strategy” to robustize the TVM exclusively into the bone marrow and delineate its mechanism. We further demonstrate that the TVM of the infused cells exceeds up to 50% of the injected cells, which till date stands at 5% worldwide. This can ameliorate the cell dose associated mortality that occurs post-radiation exposure by 100%.

It is known that regulating the TVM of particulate or cell selectively into the bone marrow compartment plays a significant role in the treatment of critical haematological disorders^20,21^. The inadequate TVM of HSCs is known to cause deleterious post-transplant mortality (PTM), which affects millions of patients and accounts for a substantial number of annual deaths globally during the treatment of various disorders. It is established that increased TVM enables atleast a few of the SCID Repopulating Cells (SRC), Long Term Culture Initiating Cells (LTC-IC), and Colony Forming Cells (CFC) that are solely essential for regeneration, enter the bone marrow, restore the number of platelets and neutrophils to threshold levels which counter the thrombocytopenia and neutropenia, and confer survival. But the conditioning regimens, such as radiation dose (3–4 Gy) or accidental radiation dose (7–8 Gy), reduce the TVM and thereby curtail the radioprotective benefits crucial for survival. The “platelet dust cluster strategy” discussed here enhances TVM of HSCs (SRCs, LTC-IC and CFCs) by very high percentage, from the low total cell dose or from minimum essential cell dose (<0.5 × 10^6^–<2.0 × 10^6^/kg bw in humans)^22–25^. This is a promising finding, particularly when this cell dose can ameliorate PTM by enabling sustainable regeneration.

## Results

### Preparation of PF127-CA HSCs and PF127-HSCBpep HSCs

**Figure 2:**
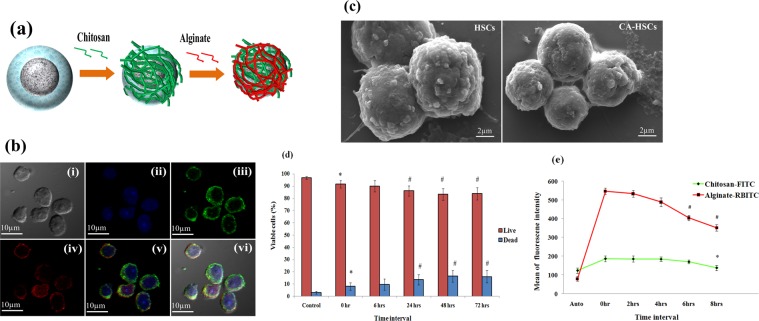
(**a**) Schematic representation of layer-by-layer technique utilized for encapsulating single cells using the polymer chitosan and alginate. (**b**) **Confocal Laser Scanning Microscopy (CLSM)**: images of the CA-HSCs (i) DIC, (ii) DAPI, (iii) chitosan-FITC layer, (iv) alginate-RBITC layer, (v) merge and (vi) DIC merge (objective: 60X, Scale: 10 µm); DAPI: 4′, 6-diamidino-2-phenylindole, UV Filter, Excitation/Emision: 358/461 nm; FITC: Fluorescein isothiocyanate, Blue filter, Excitation/Emission: 490/525 nm; RBITC: Rhodamine-β-isothiocyanate, Green Filter, Excitation/Emission: 543/569 nm. **(c) Scanning electron microscopy (SEM)**: images of HSCs and CA-HSCs (Scale: 2 µm). (**d**) Bar graph representing the results of the calcein-AM/ethidium homodimer-1 live/dead assay (*n* = 3, results as mean ± SD, *P ≤ 0.05, ^#^*P* ≤ 0.01). (**e**) **Template stability studies**: line graph obtained after incubation of templated cells in 100% serum at different time intervals. CA-HSCs were incubated in 100% serum for 2, 4, 6 and 8 hrs to access the degradation. Cells started to lose their template after six hour (*(n* = 3, results as mean ± SD,*P ≤ 0.05, ^#^*P* ≤ 0.01).

**Figure 3:**
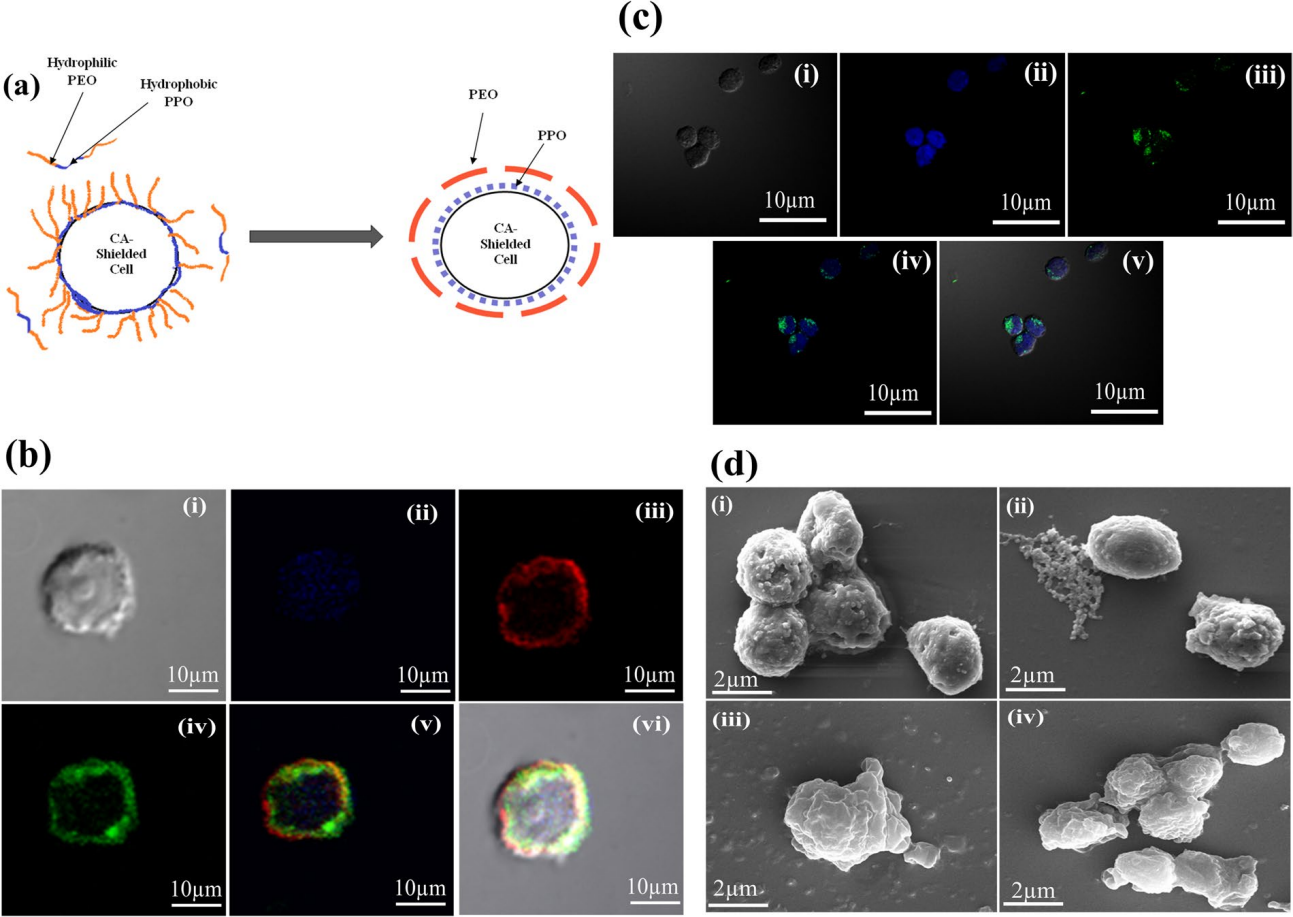
(**a**) Diagram representing binding of PF127 to the CA-HSCs **(b**) **CLSM**: images of PF127-CA-HSCs (i) DIC, (ii) DAPI, (iii) alginate-RBITC (iv) PF127-FITC layer, (v) merged image and (vi) DIC merge (objective: 60X, Scale: 10 µm). **(c) CLSM**: images of PF127-HSCBpep HSCs: (i) DIC, (ii) DAPI, (iii) PF127-HSCBpep-FITC layer, (iv) merged image and (v) DIC merge (objective: 60X, Scale: 10 µm;. DAPI: 4′, 6-diamidino-2-phenylindole, UV Filter, Excitation/Emmision: 358/461 nm, FITC: Fluorescein isothiocyanate, Blue filter, Excitation/Emission: 490/525 nm. **(d) SEM**: images: (i) HSCs, (ii) CA-HSCs, (iii) PF127-CA-HSCs and (iv) PF127-HSCBpep HSCs (Scale: 2 µm).

We enclosed the cells using two highly biocompatible biopolymers, chitosan (C) and alginate (A), to form a template for PF127 and a shield to prevent the accessing circulating cytokines. These molecules can otherwise stimulate the naked naive cells and inversely regulate natural TVM and post-TVM self-renewal of cells in the niche to preserve their stemness^26,27^. Because C (positively charged) and A (negatively charged) are expected to form strong electrostatic complexes, we built this shield by sequential layer-by-layer deposition (CA complex) (Fig. 2(a)). We confirmed the formation of the shield with confocal laser scanning microscopy (CLSM; Fig. 2(b)) and scanning electron microscopy (SEM; Fig. 2(c)). The shield maintained the integrity and viability of cells at a non-toxic concentration (1 mg/ml; Fig. 2(d) and Supplementary Fig. 1). After establishing the stable presence of the shield for up to 8 hrs (Fig. 2(e) and Supplementary Fig. 2) and thereby completely preventing the binding of the cytokines to the cells **(**Supplementary Fig. 3), the PF127 layer was grafted (hereafter referred to as PF127-CA HSCs) at a non-toxic concentration (2 mg/ml), (Supplementary Fig. 4(a)) which was confirmed by CLSM (Fig. 3(a,b)).

To assess whether the accessibility of the infused cells to the cytokines affects either TVM or the subsequent regeneration, we chose to covalently conjugate PF127 only with a specific haematopoietic stem-cell-binding peptide (STFTKSP)^28^ at a ratio of 10:1 (henceforth referred to as PF127–HSCBpep) and achieve enclosure of the HSCs by allowing their direct binding to the cells (Supplementary Fig. 4(b)). This conjugate was also investigated concurrently for its ability to mask cells (Supplementary Fig. (c)) and facilitate infusion. The binding of the PF127 and its peptide conjugate to the cell surface at non-toxic concentrations (upto 200 µg/ml) was confirmed by CLSM and SEM **(**Fig. 3(c,d)**)**.

**Figure 4:**
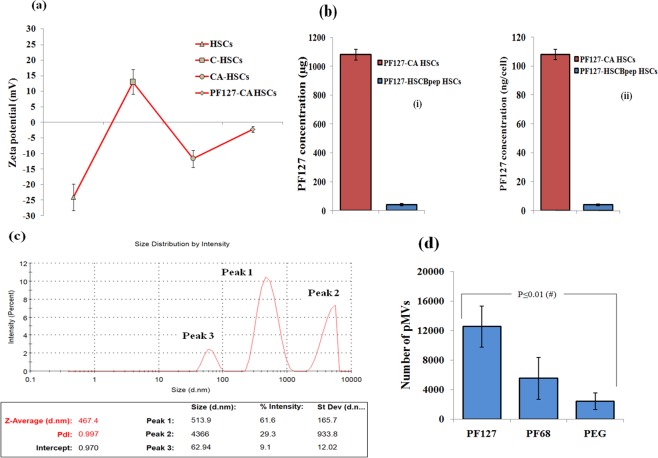
(**a**) **Zeta potential measurement**: Surface charge was measured after sequential layering of Chitosan, Alginate and PF127 on HSCs. (**b**) Bar Graph representing the quantity of PF127 grafted on the HSCs after coating with PF127-CA and PF127-HSCBpep measured by Cobalt thiocyanate assay; (i) quantity in micrograms per 10,000 cells and (ii) quantity in nanograms per cell (*n* = 3, results as mean ± SD).**(c) Size of pMVs was determined by Dynamic Light Scattering (DLS)**: Three peaks and polydispersity (PDi) value of 0.9 suggest that pMVs formed have very broad size distribution. **(d**) Bar graph representing the results of 96-well pMVs binding assay. The results suggest that out of PF127, PF68, and PEG/PEO, the higher numbers of pMVs were found attached to the well coated with PF127. (*n* = 3, results as mean ± SD).

Besides, we measured the surface charge of the PF127-CA HSCs. Changes were noticed in the surface charge after each layering; the addition of each polymer layer onto the cell suspension resulted in a shift in surface charge, which was measured as zeta potential. The charge on the HSCs was −24.02 ± 4.2 mV, which shifted to +12.99 ± 3.9 mV (p ≤ 0.01) after the deposition of the chitosan, suggesting the formation of a cationic layer over the cell surface. The charge again shifted towards a more negative value, i.e.−11.6 ± 2.7 mV (p ≤ 0.05), after anionic alginate was layered over the cells pre-coated with chitosan, producing an overall negative charge. When these CA-HSCs were treated with PF127, the charge was found to shift towards zero, i.e.−2.22 ± 0.9 mV, which confirmed the formation of the non-ionic PF127 layer over the CA-HSCs (Fig. 4(a)).

PF127 layered over the CA-HSCs was also quantitatively estimated. Here, the PF127 in PF127-CA and PF127-HSCBpep HSCs was first released by degradation through serum incubation. The quantity of the released PF127 was measured through cobalt thiocyanate assay^11,29^ using the standard curve (Supplementary Fig. 6). The quantity of PF127 taken up was found to be 1080 ± 36 µg (53 ± 1.8%) and 41.33 ± 6.4 µg (82.6 ± 12.8%) for 10,000 PF127-CA and PF127-HSCBpep HSCs, respectively, which corresponds to 108 ± 3.6 ng and 4 ± 0.6 ng PF127 per cell (Fig. 4(b-i,ii)).

When these PF127-CA HSCs were treated with pMVs or serum, a structure resembling a rotavirus was observed (nanocloud).

### Revealing the pMV sequestration potential of PF127

**Figure 5:**
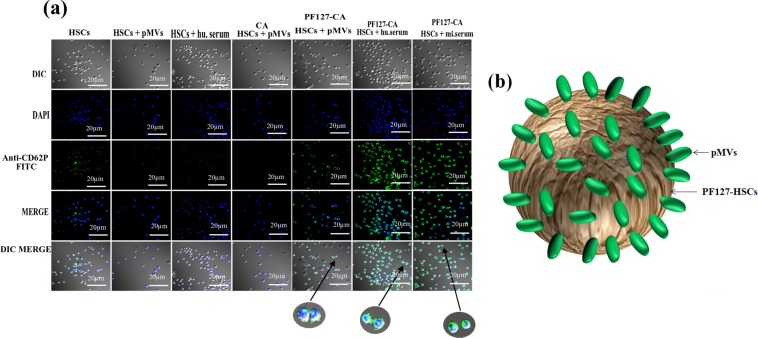
(**a**) **CLSM**: images showing pMVs binding to HSCs and PF127-CA HSCs. The binding of more P-selectin (CD62P) antibody signifies the presence of pMVs on the surface of the PF127-CA HSCs, which demonstrates the role of PF127 as a pMV accumulator. Antibody binding was even more pronounced in both human and mouse serum, as the concentration of pMVs is reported to be 10 times higher in serum (objective: 20X, Scale: 10 µm); DAPI: 4′, 6-diamidino-2-phenylindole, UV Filter, Excitation/Emission: 358/461 nm; FITC: Fluorescein isothiocyanate, Blue filter, Excitation/Emission: 490/525 nm. (**b**) Schematic representation of the presumed structure formed after attachment of pMVs to PF127-HSCs. The resulting structure may resemble like rotavirus with attached capsid spikes.

An *in vitro* binding assay of pMVs with immobilized PF127, PF68 (Poloxamer-188), and PEO (PEG) confirmed that PF127 was capable of binding to pMVs (size: Z-Avg 467.4 nm; zeta potential:−8.92 ± 1.23 mV) (Fig. 4(c)). This binding was higher in comparison with PF68 and PEO, revealing the variation in their EO/PO contents (Fig. 4(d)). Besides, we discerned that PF127-CA HSCs were able to sequester these pMVs from the human and mouse serum and accumulate them around their surface. This observation was confirmed by CLSM through enhanced binding of anti-CD62P (P-Selectin) antibody, where a structure resembling a rotavirus assembly with capsid spike proteins (like nanocloud) was observed. (Fig. 5(a,b) respectively).

### Bone marrow-specific TVM

**Figure 6:**
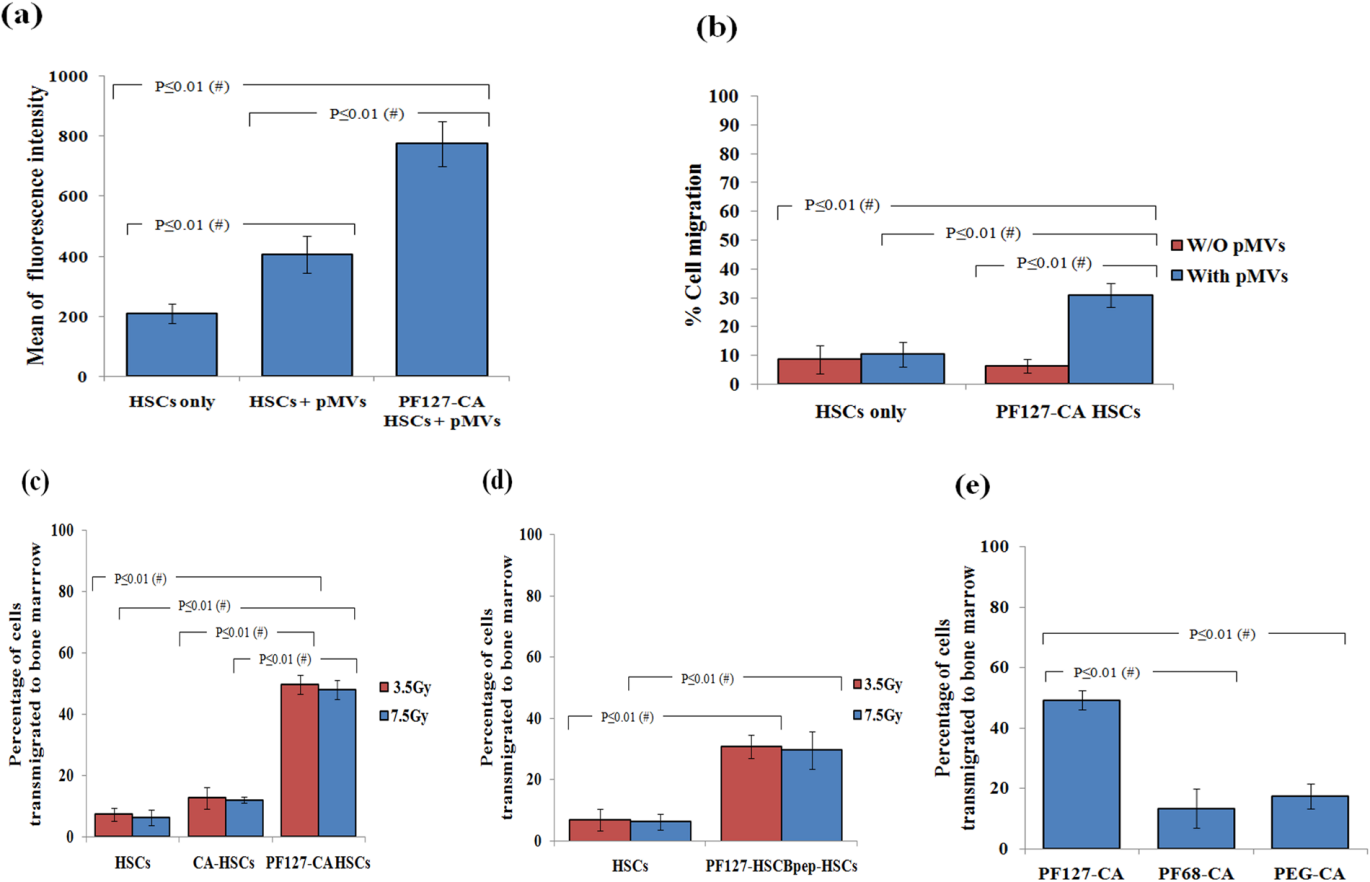
(**a**) **Flow cytometry**: Bar graph representing the expression levels of CXCR4 on pMVs treated PF127-CA HSCs and HSCs. (*n* = 3, results as mean ± SD). (**b**) Bar graph representing the results of TVM assay conducted by using human bone marrow endothelial cells and PF127-CA HSCs. (*n* = 3, results as mean ± SD). (**c**) **Flow cytometry**: Bar graph representing the results of *in vivo* TVM experiments obtained revealing the percentages of PKH26 labeled PF127-CA HSCs transmigrated to the nude mice bone marrow after 24 hrs of intravenous infusion (*n* = 6, results as mean ± SD) (**d**) **Flow cytometry**: Bar graph representing the results of *in vivo* TVM experiments revealing the percentages of PKH26 labeled PF127-HSCBpep HSCs transmigrated to the nude mice bone marrow after 24 hrs of intravenous infusion (*n* = 6, results as mean ± SD). (**e**) **Flow cytometry**: Bar graph comparing the specificity of PF127 with other triblock copolymers (PF68) and long circulating compound (PEG/PEO) (*n* = 6, results as mean ± SD).

We quantitatively assessed the level of CXCR4 in the pMVs-bound HSCs as it plays a vital role in the TVM of cells. Indeed, we identified that the levels of CXCR4 were significantly higher in PF127-CA HSCs than in HSCs only (P ≤ 0.01) (Fig. 6(a)). These increased surface-anchored levels of CXCR4 may be one of the contributing factors in enhancing its interaction with the local chemokine SDF-1 gradient built and accumulated around the bone marrow vasculature and leading to the unusual TVM response. This was firmly established by a TVM assay carried out by using human bone marrow endothelial cells cultured on insert. HSCs and PF127-CA HSCs were treated with isolated pMVs and seeded onto the insert plate. A relatively high percentage of PF127-CA HSCs (30.9 ± 4%) was found to adhere and transmigrate through the human bone marrow endothelial cell layer than in the HSCs only (10.4 ± 4.3%) (*P* ≤ 0.01). The PF127-CA HSCs and HSCs which were not treated with pMVs did not exhibit an effective TVM (6.4 ± 4.9% and 8.6 ± 2.3% respectively) (Fig. 6(b)). It thus confirms the interactions of pMVs to an extent of firm adhesion mediated by their associated molecules such as the platelet-endothelium attachment receptors GPIIb/IIIa, integrin β3, and GPIa/IIa. Hence, we speculated the combined role of both pMVs and PF127, i.e. pMVs in TVM and PF127 as an accumulator of pMVs, in bone-marrow specific TVM.

### Effect of PF127 on *in vivo* bone marrow TVM

Having ascertained that the nanocomplexes were stable and that they promoted TVM under *in vitro* conditions, we further attempted to test them under *in vivo* settings. We prepared PKH26 dye-labeled PF127-CA HSCs. These cells were injected intravenously at a dose of 1.6 × 10^4^ cells per nude mice that had received prior exposure to a conditioning dose of 3.5 Gy or a lethal dose of 7.5 Gy by total body irradiation (TBI) 24 hrs before infusion. As speculated, among the mice exposed to the conditioning or lethal radiation doses, 49.3 ± 3% (*P* ≤ 0.01) and 48.9 ± 3.05% (*P* ≤ 0.01) of the PF127-CA HSCs transmigrated specifically to the bone marrow after 24 hrs, respectively. In contrast, only 7 ± 3.6% (*P* ≤ 0.05) and 6.3 ± 2.5% (*P* ≤ 0.05) of HSCs (control group) transmigrated to the bone marrow of mice exposed to 3.5 Gy and 7.5 Gy radiation doses, respectively. To establish whether only the PF127 is responsible for this increase in TVM or the CA complex also contributes to the effect, we separately assessed cells only with the CA complex (devoid of PF127). We observed that the increase previously noted in the transmigration relative to control HSCs was reduced to 4.16 ± 3.02 (*P* ≤ 0.05) and 4.08 ± 3 (*P* ≤ 0.05) for the lethal and conditioning doses, respectively (Fig. 6(c)). This suggests that although stemness was preserved, specific TVM to the bone marrow was not achieved, thereby confirming the role of PF127 in this phenomenon.

Subsequently, to check whether PF127 is exclusively responsible for the TVM of cells to the bone marrow, we infused PF127–HSCBpep HSCs. Again, 30.88 ± 3.7% (*P* ≤ 0.05) and 29.8 ± 6.04% (*P* ≤ 0.05) of the infused cells were found to be transmigrated to the bone marrow in mice exposed to the conditioning and lethal radiation doses, respectively, when compared with the control mice receiving only HSCs (Fig. 6(d)). However, the hike was still not quantitatively similar to that of PF127-CA HSCs. Thus, the difference between the abilities of the PF127–CA and PF127–HSCBpep was appreciable (Fig. 6(c) vs (d)). Therefore, we assume that the peptide nanocomplex achieved only partial wrapping and was unable to form an effective nanocloud of pMVs, which resulted in quick release or partial/full exposure of the cells to cytokines (Supplementary Fig. 5). The combination of these two factors appears to have caused the loss of stemness and reduction in TVM.

When compared with PF127, the TVM abilities of other pluronics such as PF68 (*P* ≤ 0.01) and long circulating compounds such as PEG (*P* ≤ 0.05) were found to be minimal, suggesting that this phenomenon is exclusively brought about by PF127 (Fig. 6(e)).

We attempted to ensure whether this robust bone marrow TVM is caused by the conventional molecular interactions between the infused subset stem cells and host sinusoidal bone marrow endothelial cells or by some other mechanism. For this purpose, using the same approach, we administered chronic myelogenous leukemia K-562 cell line (CCL-243) as a non-stem cell model that does not display interactions similar to the HSCs. The cell-line was pre-labeled with characterized dextran-coated iron oxide nanoparticles (Fe_2_O_3_, 13 nm; Supplementary Figs. 7.1–7.3)^30^ and a “whole body bio-distribution study” was performed. The PF127-CA cells were injected into nude mice which were exposed to the conditioning dose. After 24 hrs, we found that the cell-bound Fe_2_O_3_ was higher in the bone marrow than in the other organs such as liver, lungs, spleen, and leg bone tissue (Supplementary Figs. 7.4–7.5). These results imply that the PF127 mechanism of robustized TVM does not depend on cell-specific molecules to guide migration across the sinusoidal vascular transendothelial checkpoints.

**Figure 7:**
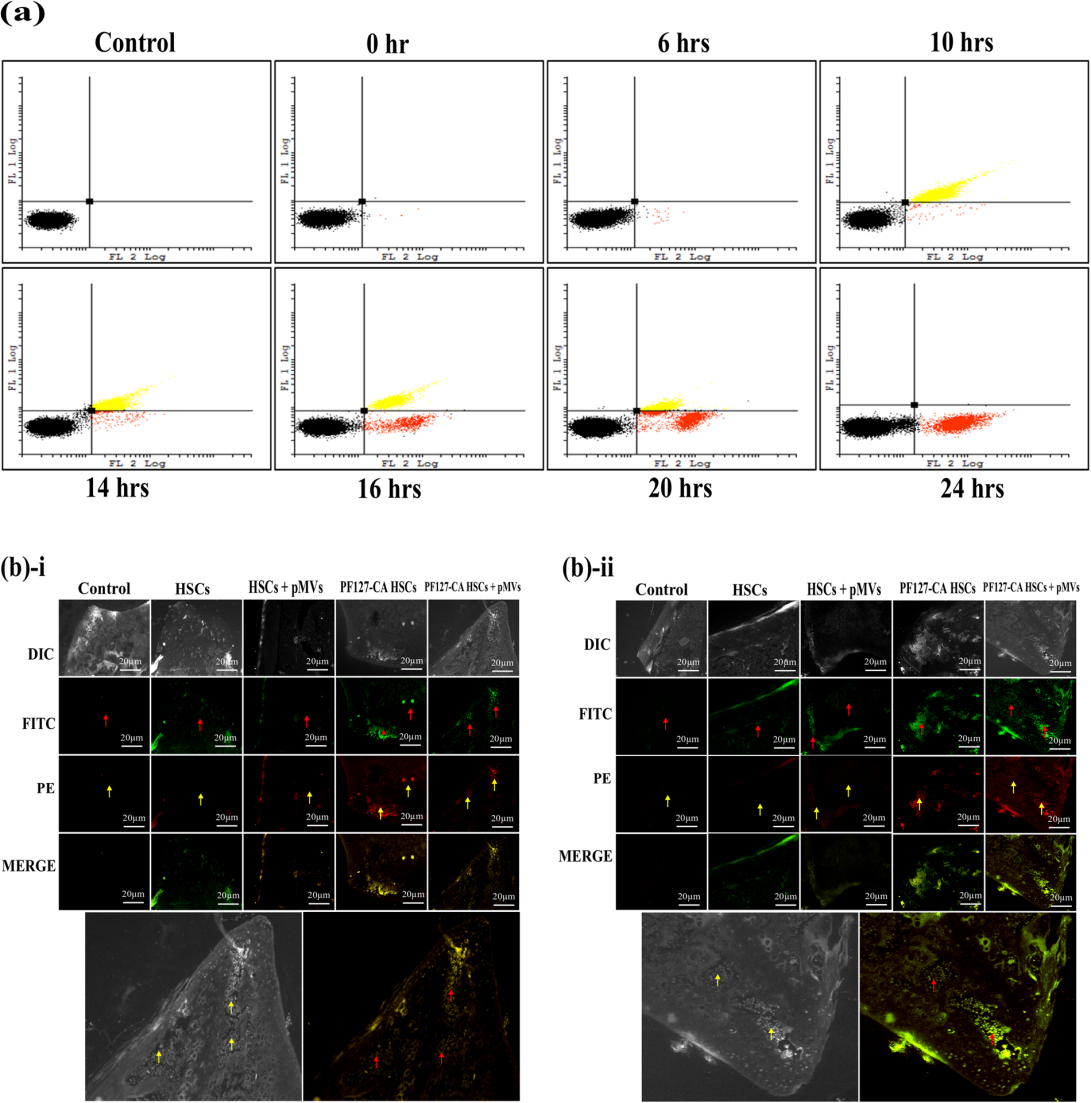
(**a**) **Flow cytometry**: TVM studies in nude mice with PKH26-labelled HSCs encapsualted with PF127 (FITC)-CA to analyze whether PF127 plays only a secondary role, i.e., only mediates the delivery of cells through the bone marrow sinusoid or travels with it. Dual-color flow cytometry analysis was performed on nude mouse bone marrow samples 0, 6, 10, 14, 16, 20 and 24 hrs after the infusion of PF127-CA HSCs. After 10 hours, most of the cells were found in the region showing dual fluorescence, i.e., upper right (yellow), suggesting that PF127-FITC travelled with the PKH26-labelled cells. However, the cells began to settle more towards the Fl-2 region, i.e., lower right (red), after 14, 16 and 20 hrs and after 24 hrs; no cells were observed in the upper right region. FL-1 = PF127-FITC; FL-2 = PKH26. (**b**) **Fluorescence microscopy**: Images obtained after bone sectioning of nude mice femur (bone marrow). Mice were infused with HSCs and PF127-CA HSCs with and without treatment pMVs after (i) 6 and (ii) 8 hrs before bone excision.

Considering these dramatic properties of the PF127 nanocomplexes, we conducted further experiments to determine whether they play only a secondary role in diverting the cells towards the bone marrow via steric hindrance caused by the PEO chains or they instead enter specifically into the bone marrow tissues along with these cells. We ruled out the first proposition because the long-circulating compounds PEO and PF68 did not display the same levels of bone marrow-specific TVM ability. Since these results implied a different mechanism involving the sinusoidal vascular endothelium in the bone marrow tissue, we infused PKH26-labeled PF127-CA HSCs in which the PF127 was directly conjugated with fluorescein isothiocyanate (FITC)^11^. Interestingly, we detected intact PF127 nanocomplexes along with cells in the bone marrow tissue after 6 hrs of infusion, which continued to persist for up to 10 hrs (Fig. 7(a)). This observation confirms that PF127 enters the bone marrow along with the cells. After a specific time interval, unwrapping happens and the cells are released into the new bone marrow niche.

Furthermore, we attempted to visualize the HSCs that managed to reach the bone marrow through histology studies. We were also interested in checking whether the pMVs move inside the bone marrow alongside PF127-CA HSCs. Microscopic examination of the bone marrow sections revealed that both PF127 (FITC) and pMVs (CD62P-PE) were able to cross the endothelial barrier along with the HSCs and reach the bone marrow compartment (Fig. 7(b-i,ii)). The deposition was primarily witnessed near the epiphysis in the cancellous (spongy) tissue where red bone marrow is located after they pass through the microvascular component created by stromal cells near the trabeculae-rich region. This vasculature further serves as a niche for haematopoiesis. We suggest that the difference in the number of cells visualized in the bone marrow is due to the potential of the PF127 nanocomplex to effectively deliver lower subset cell doses (1 × 10^4^ cells per mouse) infused at similar ratios, as observed in the case of homing with 1.6 × 10^4^ cells per mice.

### Involvement of CXCR4/SDF-1 axis

**Figure 8:**
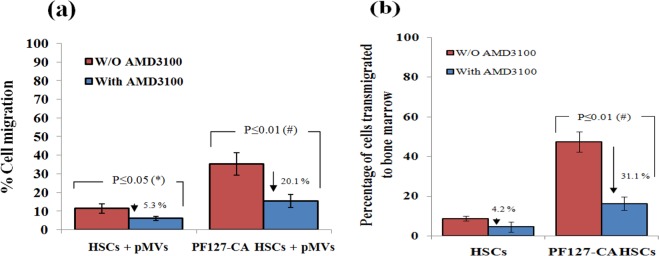
(**a**) Bar graph representing the results showing the effects of CXCR4 antagonist AMD3100 on TVM of cells. AMD3100 treated PF127-CA HSCs showed relatively less migration of cells across BMEC layer (*n* = 3, results as mean ± SD). (**b**) **Flow cytometry**: Bar representing the results of *in vivo* TVM after infusion of PF127-CA HSCs in nude mice which were treated with AMD3100 (*n* = 6, results as mean ± SD).

As the PF127-CA HSCs were found to have a high CXCR4 content, we presumed that CXCR4/SDF-1 axis may be involved in the enhanced TVM. Earlier studies have shown that pMVs carry the CXCR4 along with them, and transfer it to the cells to which they bind. Janowska-Wieczorek proved that the binding of pMVs-treated HSCs with HUVEC and immobilized SDF-1 involves CXCR4. Similarly, we found a high CXCR4 content on the PF127 layer (due to clustered pMVs) of PF127-CA HSCs. We have proven here that the TVM of PF127-CA HSCs indeed involves this axis by pre-treatment with antagonist AMD3100 (Supplemetary Fig. 10), which inhibits the binding of SDF-1 with CXCR4. Both HSCs and PF127-CA HSCs were treated with pMVs followed by AMD3100. We found that the percentage of cell migration was significantly reduced in the PF127-CA HSCs after AMD3100 treatment (15.3 ± 3.5%) in comparison with the non-treated PF127-CA HSCs (35.4 ± 6% vs. 20.1 ± 2.5%, *P* ≤ 0.01) (Fig. 8(a)).

Moreover, we performed experiments to determine whether AMD3100 could also inhibit the *in vivo* TVM of PF127-CA HSCs cell in nude mice. HSCs and PF127-CA HSCs were intravenously infused in non-AMD3100 pre-treated and AMD3100 pre-treated nude mice. After 24 hrs, we observed that the percentage of TVM in the non-AMD3100 pre-treated and AMD3100 pre-treated PF127-CA HSCs were 47.3 ± 5.5% and 16.2 ± 3.3%, respectively (*P* ≤ 0.01). Hence, we can conclude that although PF127-CA HSCs are able to sequester pMVs, a CXCR4-SDF-1 axis could not be established and TVM could not take place because of the presence of AMD3100 in the pre-treated mice. This result further confirms that the CXCR4/SDF-1 axis plays a key role in pMVs-mediated robust homing of the cells (Fig. 8(b)).

### Effect of the exorbitant TVM of cells in regeneration

**Figure 9:**
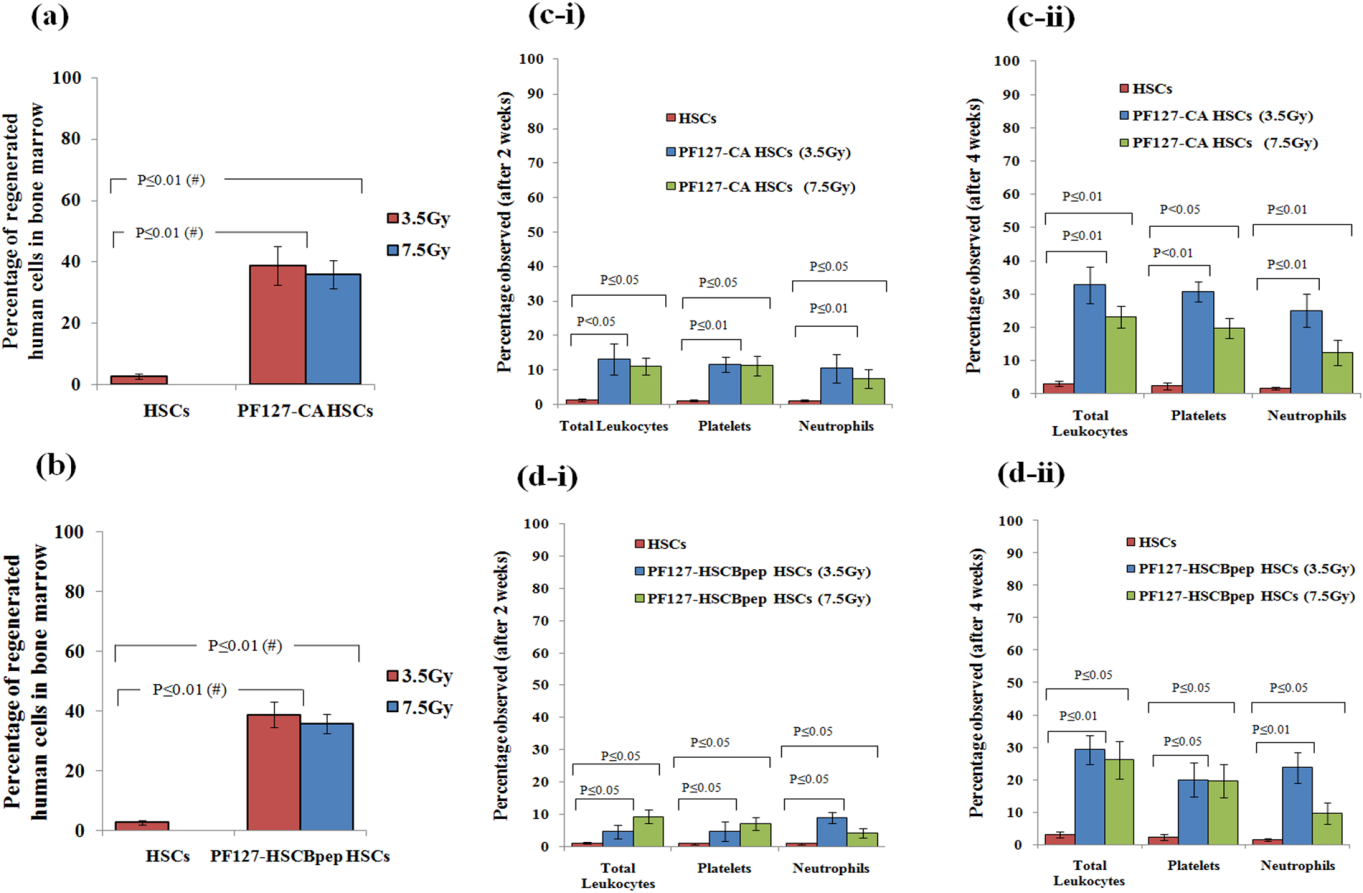
(**a**) **Flow cytometry**: Bar graph revealing the percentage of regenerated human cells detected by using anti-human mitochondria antibody in the nude mice bone marrow after two weeks of intravenous infusion of PF127-CA HSCs after conditioning (3.5 Gy) & lethal (7.5 Gy) dose (*n* = 6, results as mean ± SD). (**b**) **Flow cytometry**: Bar graph revealing the percentage of regenerated human cells detected by using anti-human mitochondria antibody in the nude mice bone marrow after two weeks of intravenous infusion of PF127-HSCBpep HSCs after conditioning (3.5 Gy) & lethal (7.5 Gy) dose (*n* = 6, results as mean ± SD). (**c**) **Flow cytometry**: Bar graphs comparing the percentage of positive human terminal lineage cells identified in the peripheral blood of nude mice using monoclonal antibodies specific for human cell surface antigens for leukocytes (huCD45), neutrophils (huCD15) and platelets (huCD41a) after (i) two weeks and (ii) four weeks of intravenous infusion of PF127-CA HSCs respectively (*n* = 6, results as mean ± SD). (**d**) **Flow cytometry**: Bar graphs comparing the percentage of positive human terminal lineage cells identified in the peripheral blood of nude mice using monoclonal antibodies specific for human cell surface antigens for leukocytes (huCD45), neutrophils (huCD15) and platelets (huCD41a) after (i) two weeks and (ii) four weeks of intravenous infusion of PF127–HSCBpep HSCs respectively (*n* = 6, results as mean ± SD).

Two weeks after the TVM of these cells into the bone marrow, regeneration was assessed using anti-human mitochondria antibody (ab92824). The percentage of regenerated cells in mice that received the conditioning radiation dose and PF127-CA HSCs was 38.4 ± 6.3% (*P* ≤ 0.01). On the other hand, in mice that received the conditioning dose and PF127–HSCBpep HSCs, the percentage was 28 ± 4.8% (*P* ≤ 0.01). Similarly, the percentage of regenerated cells in mice exposed to the lethal dose and similar treatment were 35.15 ± 4% (*P* ≤ 0.01) and 24.7 ± 6.9% (*P* ≤ 0.01), respectively **(**Fig. 9(a,b)).

To investigate the successful differentiation, we subsequently explored the time reduction to discern the number of platelets required for non-thrombocytopenic (huCD41a^+^) condition and the number of neutrophils needed for non-neutropenic (huCD15^+^) condition. After two weeks, the regenerated platelets and neutrophils were 11.46 ± 4.1% (*P* ≤ 0.01) and 10.43 ± 2.1% (*P* ≤ 0.01), respectively, for the conditioning dose in the case of PF127–CA HSCs. In the fourth week, their numbers increased to 30.66 ± 5% (*P* ≤ 0.01) and 25 ± 3.5% (*P* ≤ 0.01), respectively. In the case of only HSCs, the percentage of regenerated cells was only 1.05 ± 0.23% (*P* ≤ 0.01) and 1.03 ± 0.24% (*P* ≤ 0.01), respectively, after the second week and 2.29 ± 1.02% (*P* ≤ 0.01) and 1.51 ± 0.34% (*P* ≤ 0.01), respectively, after the fourth week. After lethal dose exposure and PF127-CA HSCs infusion, the respective percentages of these cells were 11.13 ± 2.7% (*P* ≤ 0.05) and 7.4 ± 2.7% (*P* ≤ 0.05) after the second week, which further increased to 19.64 ± 5% (*P* ≤ 0.05) and 12.25 ± 3.5% (*P* ≤ 0.01) after the fourth week (Fig. 9(c-i,ii)).

Although we found comparable trends in PF127–HSCBpep HSCs, the results were not similar to those of PF127–CA HSCs. For example, in the case of conditioning dose, the percentages of regeneration were 4.9 ± 1.8% (*P* ≤ 0.05) and 9 ± 3% (*P* ≤ 0.05), respectively, after the second week, and they increased to 20 ± 5.2% (*P* ≤ 0.05) and 23.8 ± 4.75% (*P* ≤ 0.01), respectively, after the fourth week in comparison with HSCs only. For the lethal dose, we found that the respective percentages after the second week were 7.1 ± 1.95% (*P* ≤ 0.05) and 4.3 ± 1.5% (*P* ≤ 0.05),and these values increased to 19.6 ± 5.1% (*P* ≤ 0.05) and 9.6 ± 3.2% (*P* ≤ 0.05) after the week (Fig. 9(d-i,ii)). This exorbitant TVM decreased the time required for the number surge necessary to avoid deficiency, which is usually 8–12 weeks in conventional settings, by 1–1.5 fold (Supplementary Table. 1).

### Effect of exorbitant TVM on survival

**Figure 10:**
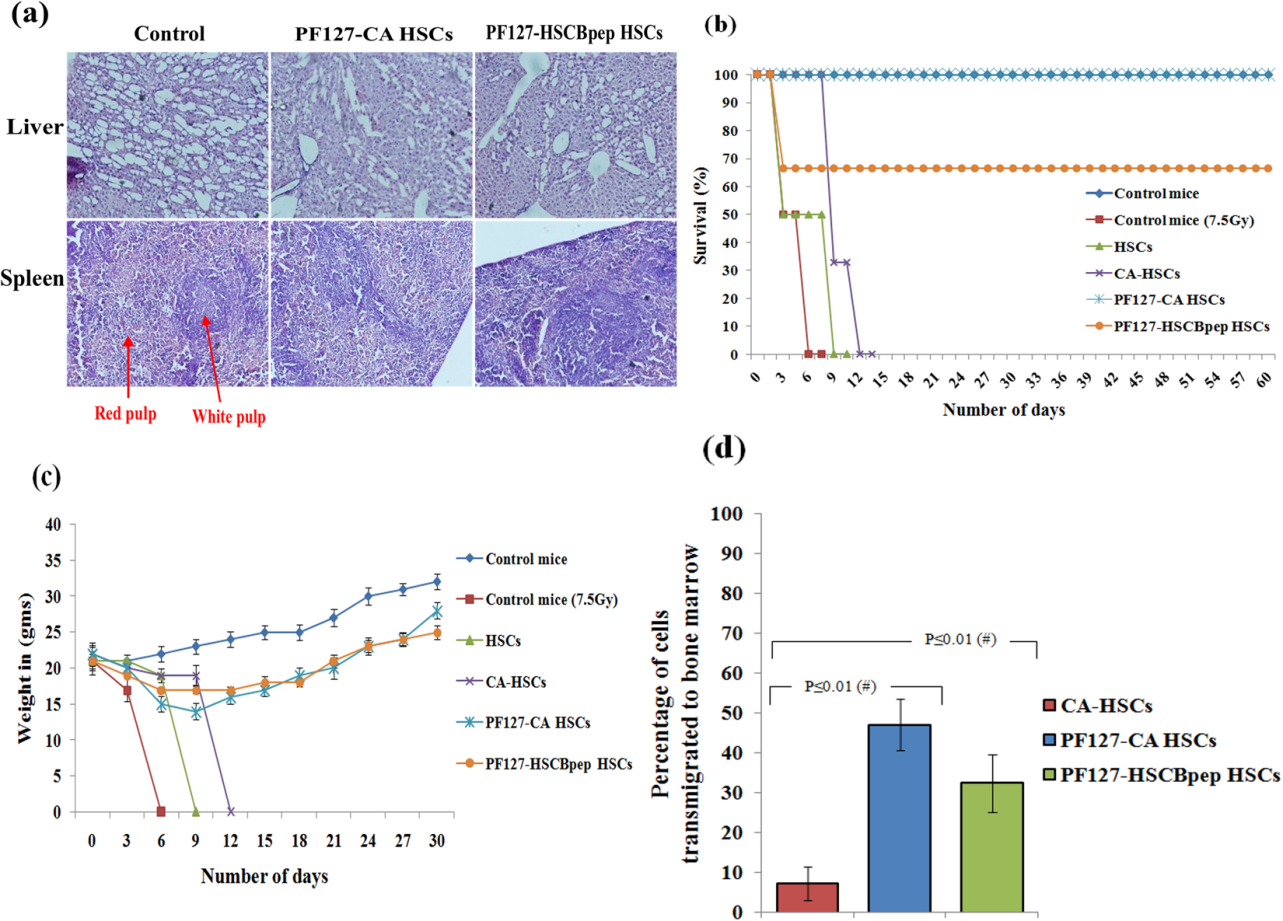
(**a**) **Histology**: microscopic examination of liver and spleen tissue after 1 week of infusion of PF127-HSCs (objective 40X). No necrosis, swelling, or degradation was detected in the hepatic cells. The splenic tissues showed normal patterns in the red pulp region and the white pulp region. Next, the weight gain and the survival of lethally irradiated (7.5 Gy) mice after infusion with treated cells was monitored. (**b**) The survival of mice was monitored for up to 60 days and **(c)** Weight was monitored up to 30 days. (**d**) **Flow Cytometry**: Bar graph revealing the percentages of PKH26-labelled PF127-CA HSCs transmigrated to immunocompromised rabbit bone marrow 24 hrs after intravenous infusion (*n* = 3, results as mean ± SD).

We observed that eventually the mouse mortality and morbidity were successfully reduced to zero, particularly after the lethal dose exposure, when the subset cells received PF127–CA HSCs during the 60 days of observation. However, similar results were not noticed in mice treated with PF127–HSCBpep HSCs or without the complete nanocomplexes. The maximum survival was only founded on the use of PF127–CA. We also observed that the bodyweight of mice that received cells with PF127–CA HSCs surged concurrently with the increase in survival. The 34% decrease in survival observed in case of PF127–HSCBpep on day 30 witnessed in the same setting implies that this nanocomplex achieved suboptimal TVM and regeneration with corresponding kinetics, which is insufficient to rescue the recipients from PTM occurring in post critical-dose radiation exposed mice. (Fig. 10(a–c)**)**

### Effect of PF127 on TVM of SRC, LTC-IC cell numbers

It is well known that cells present in extremely low-frequency but having long-term regeneration potential, such as SRCs and LTC-ICs, and short-term multi-lineage progenitor cells, such as the progressed LTC-ICs and CFCs, are actually responsible for ensuring survival through regeneration. Therefore, the significant enhancements in survival, bone marrow regeneration, progenitor and innate cell development (collectively known as sustained engraftment) observed in the case of PF127–CA are due to its extremely high conduct of TVM into the bone marrow. In a study by Bhatia *et al*., the results of an authentic serial dilution assay revealed that the numbers of SRCs and LTC-ICs in the cell subset are 1 per 617 cells and 20–100 per 1000, respectively^31^. By considering the reliability of these reported values, we computed the proportions in the experimental cell subset treated with PF127–CA. Substantial numbers were found to transmigrate to the bone marrow (Table 1).

**Table 1:** Analysis and computation of number of subset cells and specific cell types likely to have transmigrated to bone marrow.

Treatment of mice		Percent total		No. of total subset cells in bone marrow		Specific cell subtypes in subset infused (in folds)				Comparison of SRC/617 and LTC-IC/10^3^ subset cells in bone marrow in different treatments (in folds)	
		CD	LD	CD	LD	SRCs		LTC-ICs			
						CD		LD			
A	Nil	—	—	—	—	—	—	—	—	SRCs	LTC-ICs
B	Only HSCs	7.0 ± 3.6	6.3 ± 2.5	1120	1008	1.8	1.6	22.4–112	20–100	B:A(CD) = 1.8 B:A(LD) = 1.6 C:B(CD) = 7.1 C:B(LD) = 7.9 D:B(CD) = 4.4 D:B(LD) = 4.8 E:B(CD) = 2.0 E:B(LD) = 1.93 D:C(CD) = 1.6 (less) D:C(LD) = 1.64 (less) C:E(CD) = 3.45 (less) C:E(LD) = 4.09 (less)	B:A(CD) = 22.4–112 B:A(LD) = 20–100 C:B(CD) = 6.96–6.96 C:B(LD) = 7.8–7.8 D:B(CD) = 4.3–4.37 D:B(LD) = 4.7–4.76 E:B(CD) = 1.7–17.85 E:B(LD) = 1.98–19.2 E:C(CD) = 4.0 (less) E:C(LD) = 3.9 (less) D:C(CD) = 1.61–15.9 (less) D:C(LD) = 1.65–1.63 (less) E:D(CD) = 2.45 (less) E:D(LD) = 2.45 (less)
C	PF127–CA HSCs	49.3 ± 3.0	48.9 ± 3.05	7888	7824	12.78	12.68	156–780	156–782		
D	PF127–HSCBpep HSCs	30.88 ± 3.7	29.8 ± 6.04	4940	4768	8.0	7.7	98–490	94–476		
E	CA-HSCs	11.85 ± 0.99	11.98 ± 1.0	2022	1920	3.7	3.1	20–200	40–192		

No. of infused cells/mouse = 16000. Cell number detected 24 hrs post infusion in bone marrow as described in text. CD: conditioning dose; LD: lethal dose; SRC: SCID-repopulating cells; LTC-IC: long-term-culture-initiating cells.

In the conditioning dose, PF127-CA must have improved the TVM of SRCs by 7.1-fold and that of LTC-ICs by 6.96-fold of the cell dose used for infusion. However, in the lethal dose, the corresponding increase must have been 7.9-fold for SRCs and 7.8-fold for LTC-ICs. The effects of the PF127–HSCBpep were 4.4-fold and 4.3–4.37-fold lower for the SRCs and LTC-ICs, respectively, in the animals exposed to the conditioning dose. In case of the lethal dose, the change was 4.8-fold for SRCs and 4.7–4.76-fold for LTC-ICs. Similarly, in the animals exposed to the conditioning dose, the tentative TVM of these specific cells prepared by using only the template CA complex (without PF127) must have increased only 3.55-fold for SRCs and 3.9-fold or 4-fold less than that of the PF127–CA nanocomplex. We therefore opine that among these subset cells, the PF127–CA assists in maximising the TVM manifold when compared with the control, and performs better than the other complexes. This formulation enabled a 12.78(13)-fold increase in the TVM of SRCs at both the radiation doses, and similarly, a 156–782-fold change in the TVM of LTC-ICs within the total subset cells that were infused.

Among the total infused cells, a minimum of four SRCs and 50–250 LTC-ICs are required to transmigrate into the bone marrow for effective tri-lineage haematopoiesis^31,32^, which is only possible with a dose of 5 × 10^4^ subset cells/kg bw or higher. These values observed in mice models cannot be accurately extrapolated to kg bw of human subjects/recipients (however, a crude estimate of <10 × 10^6^/kg bw has been generated for the initial cell threshold in clinical human infusions.), with which we made an intra-comparison when different nanoformulations were used with control in our study. We concluded that the TVM of these cells by the PF127–CA is many fold higher. The extended survival of mice, particularly seen in this case and on lethal dose exposure, is therefore likely to be the result of accelerated TVM kinetics by PF127-CA and sustained subsequent regenerative processes. Nonetheless, the percentages of “transmigrated” out of the total infused HSCs dose treated with PF127–CA were 49.3% and 48.9% for the two radiation doses, contrary to the 5% that has been reported in the literature till date. Our findings of 9.78 and 9.78-fold of the total initial cell dose infused in each case is the highest among the other nanoformulations. The relative surge in the percentage generation of leukocytes, platelets, and neutrophils confirms that this formulation along with infused cells holds the potential for effective treatment. Any renewed stem cells native to the surviving mice upon exposure to both radiation doses might also have relatively contributed to the observed survival.

### Effect of PF127 on TVM in rabbits

To address the validity of these results in a higher animal model and to account for variations in the bone marrow vasuclar endothelium surface, homing experiments were performed using rabbits as they are phylogenetically similar and their bone marrow has a sinusoidal ultrastructure akin to that of humans^33,34^. Since the ^60^Co irradiators available worldwide do not accommodate whole rabbits, we could not perform TBI of these animals. Instead, we used unirradiated and cyclophosphamide-immunosuppressed rabbits and performed assessment similar to the ones conducted in nude mice using PF127-CA HSCs. The PF127–CA nanocomplex achieved a comparable degree of TVM to the bone marrow of these rabbits at high doses of 47 ± 6.4%. This value is 39.7 ± 9.8% higher than the dose achieved by the CA complex without PF127, which was only 7.3 ± 4.3% (a 6.4-fold improvement; *P* ≤ 0.05; Fig. 10(d)). In contrast, the nanocomplex PF127–HSCBpep enabled the TVM of these cells at a dose of 32 ± 7.2%, which was 14.6 ± 4.4% lesser than the value obtained with the complete nanocomplex (*P* ≤ 0.05; 1.25-fold less). However, the transmigrated dose was still higher by 25.09 ± 9.8% than that of the subset cells with only the CA template and lacking PF127 (*P* ≤ 0.05).

## Discussion

To achieve the targeted delivery of a molecule, such as a drug to a specific site, the loaded nanoparticles need to first escape recognition by the reticuloendothelial system (RES). Besides, these nanoparticles need to have adsorbed a protein on their surface, which enables them to mediate the uptake by cells. In one instance, targeting to the brain was achieved with polysorbate-80 stabilized polymeric nanoparticles using apolipoprotein E (apoE) as the mediating component^35,36^. Similarly, efficient surface modifiers, such as PEO containing nonionic triblock copolymers that are also known as pluronics, can be used to reduce the RES uptake. In one study, apoE was found to preferentially adsorb on solid lipid nanoparticles (SLN) coated with pluronics having a low number of PEO units (poloxamer 184 and poloxamer235)^9^, which is in agreement with the findings of Blunk^37^. The SLN stabilized with polymers containing long PEO chains (especially poloxamine 908, poloxamer 338, and poloxamer 407) simultaneously exhibited pronounced adsorption of fibrinogen (Fbn).

As mentioned earlier, a few contradicting studies have suggested that pluronics with long PEO chains act as an Fbn resistant material and tend to minimize or inhibit Fbn binding and thereby avoid platelet aggregation and fibrin formation^38^. This, however, is not always completely true according to certain contemporary studies. In contrast to the PEO chains that are either too long or too short, those with optimal MW were observed to be more capable of interacting with proteins and inducing conformational changes through more stable binding sites and stronger interactions. For instance, ^1^H-NMR data indicate that a PEO can bind to hydrophobic pockets formed by Arg61, Trp62, Trp63, Arg73, Lys96, and Asp101 in lysozyme^39^. It was also noted that PEO tends to enclose and extensively coordinate the positively charged amino acids lysine, arginine and histidine residues and form linear binding patterns in the clefts of the protein surface. The predominant contacts are hydrogen bonds with the amino acid side chains acting as hydrogen donors and the main-chain NH groups. Another very specific type of interaction is the coordinated embedding of the cation from the solution, forming hydrophobic contacts between the outer envelope of the complex cation and the prevailing hydrophobic areas on the protein surface.

The observation of Porter *et al*. about the accumulation of PF127-treated colloidal particulates in the bone marrow after the intravenous administration in rabbits^40^ kindled our curiosity. It was also observed that colloids were sequestered by the sinusoidal endothelium instead of the macrophages. Interestingly, this phenomenon was not seen in other copolymers that were similar in structure and MW to PF127. However, it was vaguely understood that the TVM of these colloids requires PF127 having an average MW of 13.3 kDa and containing 84.6 mol% PEO^41^. Blunk in his study also related the bone marrow transvascular deposition of PF127-coated polystyrene microparticles to the chain length and MW. Being of optimal MW and having an intermediated chain length, they were able to absorb certain proteins, which resulted in their TVM in the bone marrow. However, others with even longer chains were found to circulate in the blood for longer periods. It is also assumed that the surface of the polymer-coated nanospheres adsorbs a critical plasma component that exhibits specificity for certain microdomains on the sinusoidal endothelial cell membrane. Although the effects of PF127 on the sinusoidal vascular endothelium are known to include the shedding of various membrane-bound proteins including surface mucins, P-selectin glycoprotein ligand-1, leukosialin, CD45RA, CD96, and other endothelial counter-receptors (vascular addressins), the exact roles of these proteins in the mechanistic basis of TVM were never clearly demonstrated.

Here we report for the first time the unique potential of PF127 to sequester pMVs present in blood or serum, thereby building a pMVs nanocloud on HSCs, which can be harnessed to achieve multifold TVM of transfused human stem cells into a new bone marrow niche. A robust TVM amounting to 50% of the total cell dose infused in appreciable numbers into the bone marrow subsequently resulted in a faster regeneration of the platelets and neutrophils, thereby overcoming the radiation-induced thrombocytopenia and neutropenia and conferring the survival of mice after lethal radiation exposure. The role of pMVs in TVM was previously explored by Janowska-Wieczorek *et al*. in a study which postulated that CD34^+^ HSCs present in the peripheral blood not only express typical antigens but also high levels of platelet-derived antigens^18^. Further, it was established that as the HSCs express P-selectin glycoprotein ligand-1 (CD162) and integrin Mac-1, they can interact with platelets in the peripheral blood. Later, it was found that pMVs and not intact platelets bound to HSCs that tend to carry some of the platelet properties such as antigens (CXCR4), platelet membrane glycoprotein CD41 (GPIIb/IIIa or integrin α_IIb_β_3_), CD61 (Integrin β3), CD62P (P-Selectin), PAR-1, GPIa/IIa, and others such as IIIa, IV, V, IX, etc., which are transferred to the cells to which they bind, thereby altering their molecular nature (molecular alteration)^42^.

We discovered that the locally-accumulated CXCR4 receptors attributed to the presence of pMVs on the PF127-complexed cells caused their firm adherence and efficient TVM through the human bone marrow endothelial cell layer. They were further found to interact through the soluble SDF-1 gradient near the bone marrow endothelial cells or the endothelial cell membrane bound SDF-1/CXCR4 receptor. This phenomenon was firmly established by using the CXCR4 antagonist AMD3100 which reduced the transendothelial migration of the pMVs pre-treated with PF127-CA HSCs and also the *in vivo* homing of PF127-CA HSCs in nude mice. Histological analysis of the bone marrow sections also revealed that PF127-bound pMVs transmigrate into the bone marrow along with HSCs, which confirms the firm interaction of pMVs with the endothelial cells.

Here, it is important to propose that PF127-mediated TVM can be both active and passive in nature. The PEO chains prevent the uptake of cells by RES, leading to their deposition near the bone marrow site. At the same time, extensive sequestration of pMVs from the circulation leads to the TVM of cells across the bone marrow. Nonetheless, the actual mechanism behind the binding of pMVs to PF127 remains to be elucidated. The potential candidates for the interaction and adhesion of pMVs to PF127 are the pMVs-derived integrins such as GPIIb/IIIa, β3, GPIa/IIa, etc. Studies suggest that pluronics can bind to the GPIIb/IIIa complex of platelets, and this might be the underlying mechanism for their interaction with pMVs.

This mechanism by which PF127 greatly enhances the TVM of infused haematopoietic subset cells to the bone marrow has not been previously reported in any model. This unusual property of an apparently non-toxic material might find application in customizing the treatment of many haematological disorders. Such methods include the transplantation of HSCs through dose alteration per kg bw, co-habiting the mesenchymal stem cells, transfusion of genetically engineered stem cells, etc. This feature offers a great advantage for the further exploration and customization of this technology for more widespread applications in humans, particularly in the treatment of severe haematological malignancies that require the use of these cells at high doses.

## Methods

### Preparation of pMVs

pMVs were prepared from platelets as described earlier^18,43^ after a few modifications. In brief, blood was collected from healthy volunteers and anticoagulated with 3.8% sodium citrate and adjusted to a final volume of 8 ml with Tyrode buffer (1 mg/ml albumin; 5 unit/ml apyrase; and 1 mM EGTA, pH 6.5). Platelet-rich plasma (PRP) was prepared from blood by centrifugation at 200 *g* for 30 mins to pellet the erythrocytes and leukocytes. The PRP was centrifuged at 1000 *g* for 30 mins at 20 °C to pellet the platelets and produce platelet-poor plasma (PPP) supernatant. The PPP was centrifuged twice at 1200 *g* for 30 mins at 22 °C to remove the remaining platelets. The concentrated platelets were resuspended in HEPES buffer (pH 7.5) and activated by incubation with 1 unit/ml thrombin (MP Biomedicals) and 2.5 mM CaCl_2_ with periodic shaking. After 10 mins incubation at 37 °C, the large platelet aggregates were sedimented at 1200 *g* for 30 mins, and the pMVs-containing supernatant was collected and diluted in PBS (pH 7.4) at a ratio of 1:5. Dynamic light scattering was used to determine the size and zeta potential of the pMVs (Zetasizer nanoS90, Malvern).

### Ethical approval

The approval from the INMAS Institutional Human Ethics Committee (Ref no. IIHEC/CT/2017/09; dated 27/10/2017) was obtained for the above work and appropriate measures were taken to follow all ethical norms throughout the study. Information describing the study was shown to participants and informed consent was obtained. All experiments were performed in accordance with the approved guidelines and regulations of Institutional Human Ethics Committee.

### Quantitative estimation of pMVs binding

A black 96-well fluorescence plate was treated with 0.01% poly-l-lysine (PLL) for 24 hrs. After PLL coating, PF127, PluronicsF68 (PF68, Poloxamer 188; MW 8.4 kDa) and PEO (PEG: MW 4.5 kDa) were added to the wells at 2 mg/ml and incubated for 2 hrs at 37 °C for the formation of the polymer layers. After removal of the polymer solutions, 50,000 human plasma-derived pMVs were added to each well (Supplementary Fig. 8) and incubated for 2 hrs at 37 °C. The pMVs-containing solution was then carefully removed and anti-human CD62P-FITC antibody (555523, BD Biosciences) was added to the wells for detecting the bound pMVs. After 30 mins of incubation, measurements were taken at the excitation/emission wavelengths of 490**/**530 nm (Varian Cary Eclipse Fluorescence Spectrophotometer, Agilent), and the bar graph was plotted using a standard curve (Supplementary Fig. 9). Each experiment was conducted in triplicates (n = 3), and the data were represented as mean ± SD.

### Cell encapsulation, stability, and permeability studies

#### Culture of human bone marrow haematopoietic stem/progenitor CD34^+^ cells

For haematopoietic stem/progenitor cell culture, a total of 1 × 10^5^ cryopreserved human bone marrow CD34^+^ haematopoietic cells (Lonza: 2M-101) were thawed and cultured overnight before use for each experiment in IMDM containing 0.2% FBS, 100 U/ml penicillin, and 100 µg/ml streptomycin inside a water-jacketed CO_2_ incubator (5% CO_2_, 95% humidity, and 37 °C).

#### Cell encapsulation

The biocompatible polymers chitosan (C) and alginate (A) were used to build a template on HSCs. Chitosan (deacetylation ≥85%; MW: 190 kDa) is a deacetylated form of chitin. This natural polymer is an amino polysaccharide and cellulose derivative found in the shells of shrimps and other crustaceans. The compound is a copolymer of N-acetyl glucosamine and β-(1 → 4) -linked D-glucosamine. Alginates (MW: 240 kDa) are polysaccharides composed of (1–4)-linked β-D-mannuronic and α-L-guluronic acids. The layer-by-layer deposition technique was used for encapsulating the cells^44–46^. The technique involves the alternate deposition of oppositely charged polymers over a negatively charged cell. In brief, 1% cationic chitosan (C) solution was prepared in 1% acetic acid. The solution was diluted to 1 mg/ml in Hank’s balanced salt solution (HBSS), added to a 1 × 10^5^ cells/ml suspension of HSCs, and allowed to interact with the negatively charged cell membrane for 5 mins. After removing the chitosan by washing with HBSS, anionic alginate (A) solution (1 mg/ml in HBSS) was added and the polymer was allowed to combine with the chitosan-coated cells for the next 5 mins, which resulted in cells enveloped by the polymer layers - one each of chitosan and alginate. These cells are referred to as “CA-CD34^+^ cells” or simply “CA-HSCs”

#### Confocal laser scanning microscopy (CLSM)

Fluorochrome labeled polymers were used to evaluate the firmness of the template. Chitosan and alginate were conjugated with Fluorescein isothiocyanate (FITC) and Rhodamine B isothiocyanate (RBITC), respectively, using the protocol as described earlier with slight modifications^47^. In brief, a 1% chitosan solution was prepared in 1% acetic acid solution, and a 1% alginate solution was prepared in water. The chitosan was labeled by the addition of 2 mg/ml FITC, and the reaction was allowed to proceed for 3 hrs with constant stirring. The labeled chitosan was then precipitated with 0.2 M NaOH. The solution was subsequently dialyzed against deionized water in the dark to remove excess FITC by changing the water once every 8 hrs, and this process was continued until there was no unreacted FITC. Similarly, RBITC (1 mg/ml in DMSO) was added to 1% alginate solution, and the reaction was allowed to proceed for 1 hr with constant stirring at 40 °C. The reaction was arrested by the addition of ethanolamine to the solution. The unconjugated RBITC was eliminated by dialysis under conditions similar to those employed for FITC. The extent of labelling was determined spectrophotometrically^47^ (Multi Skan Go, UV-Vis Spectrophotometer, Thermo Fisher Scientific). Subsequently, the degree of substitution was estimated at 490 nm to be 0.9 for chitosan (9 molecules per 10 sugar units) and0.06 for alginate (6 molecules per 100 sugar units). The encapsulated cells were fixed in 4% (w/v) paraformaldehyde (PFA) solution for 10 mins at room temperature. The cells were subsequently washed twice with HBSS and observed under a confocal microscope (Olympus Fluoview FV1000).

#### Scanning electron microscopy (SEM)

The surface morphology of the multilayer encapsulated CA-HSCs was studied with scanning electron microscopy. A droplet of cell suspension was placed on a glass slide that had previously been attached to a metallic stub (standard pin mount, aluminium, grooved edge) with bi-adhesive carbon tape. The slide was air-dried and further coated with gold particles to provide a conducting surface. Sample analysis was performed with a scanning electron microscope (Zeiss EVO40, Carl Zeiss) operated in vacuum mode.

#### Cell viability analysis

An assay was performed to check for the toxic effects of the procedure on the cells. Cell viability was evaluated using the LIVE/DEAD Viability/Cytotoxicity Kit (Thermo Fisher Scientific). In this assay, the intracellular esterase activity determines the conversion of non-fluorescent cell-permeant calcein-AM to intense fluorescent calcein. The dye is retained within the live cells, producing intense green fluorescence (Ex/Em wavelengths: 495/515 nm). Ethidium homodimer-1 (EthD-1) enters the cells with damaged membranes and undergoes a 40-fold enhancement upon binding to nucleic acids, thereby producing a bright red fluorescence in dead cells (Ex/Em wavelengths: 560/635 nm).

EthD-1 is excluded by the intact plasma membrane of live cells. Non-encapsulated cells were used as positive controls (viable cells), and 0.1% Triton X-100 treated cells were used as negative controls (dead cells). The fluorescence emissions were measured separately for calcein-AM at 530 nm and ethidium homodimer-1 at 645 nm (Varian Cary Eclipse Fluorescence Spectrophotometer, Agilent). Cell viability was analysed at 0, 24, 48 and 72 hrs after shielding in culture. Each experiment was conducted in triplicates (n = 3), and the data were represented as mean ± SD.

$${\rm{Percentage}}\,{\rm{viability}}( \% )=\frac{{\rm{Absorbance}}\,{\rm{of}}\,{\rm{treated}}\,{\rm{cells}}}{{\rm{Absorbance}}\,{\rm{of}}\,{\rm{control}}\,{\rm{cells}}}\times 100$$

#### Template stability studies

The cells were incubated with 100% serum as it contains enzymes that can degrade the layers. The cells were encapsulated using Chitosan-FITC and Alginate-RBITC and then incubated. The serum was removed every 2 hrs, and the cells were fixed. Later, the cell-associated fluorescence was measured using a flow cytometer (BD FACS Calibur, USA). Any reduction in fluorescence was regarded as degradation of the layers. Each experiment was in triplicates (n = 3), and the data were represented as mean ± SD. The incubated cells were also observed under a confocal microscope for corroboration (Olympus Fluoview FV1000).

#### Cytokine permeability assay

Four cytokines most likely to affect the proliferation of stem cells, namely Flt-3 ligand (17.6 kDa), SCF (18.6 kDa), IL-3 (15 kDa), and GM-CSF (16 kDa), were chosen for analysis. The template of fluorescent polymers was made on the cells. Later, 1 × 10^5^ cells/ml was incubated with the respective cytokines for 30 mins at appropriate dilutions followed by washing with HBSS. Subsequently, these cells were incubated with 100% serum to dissociate the layers, and the presence of cell-bound cytokines which had crossed the layers was checked using anti-cytokine antibodies. The samples were assessed with a flow cytometer (BD FACS Calibur), and the mean fluorescence intensity was measured. Each experiment was conducted in triplicates (n = 3), and the data were represented as mean ± SD.

### PF127 layering and cell viability assessment

#### PF127 layering on CA-HSCs

A final layer of PF127 (MW: 12.6 kDa) was applied to CA-HSCs. For viability assay, PF127 solutions of different concentrations (0.5, 1, 2, 3, 4 and 5 mg/ml) were prepared in water. Twenty microliters of the solution was added to the cell suspension of 1 × 10^5^ huCD34^+^cells/ml and left for 5 mins. Toxicity was evaluated using a 3-(4, 5-dimethylthiazol-2-yl)-2, 5-diphenyltetrazolium bromide (MTT) assay. In this protocol, MTT was added to each well at a final concentration of 0.5 mg/ml and incubated for 3 hrs. The absorbance of the formazan product was then measured at 570 nm. The cells are hereafter referred to as “PF127–CA HSCs”. Each experiment was conducted in triplicates (n = 3), and the data were represented as mean ± SD. For CLSM, PF127 labeled with FITC was used^11^. After the formation of nanocomplex, the cells were fixed in 4% (w/v) PFA solution for 10 mins at 4 °C, washed twice with HBSS, and observed under a confocal microscope (Olympus Fluoview FV1000).

#### PF127-HSCBpep layering/binding

An approach not involving the intermediate CA template and enabling the direct binding of PF127 to the HSCs was also simultaneously developed. The haematopoietic stem-cell-binding peptide (HSCBpep) was directly conjugated with PF127 and allowed to interact directly with the HSCs to form a protective coating. The heptapeptide sequence “STFTKSP”, which has the potential to bind human HSCs, was selected from the studies of Nowakowski *et al*.^28^.

The peptides were synthesized (by Link Biotech, India) using standard FMOC chemistry based on the solid phase methodology. This method utilizes base labile FMOC (Fluorenylmethyloxycarbonyl chloride) N-terminal and side chain protection as well as an acid-labile resin linkage. This step is followed by the final acidic cleavage using trifluoroacetic acid (TFA) to release the assembled peptide chain from the solid support. The synthesized peptide was precipitated, lyophilized, purified by HPLC, re-lyophilized, and characterized using a mass spectrometer (TSQ Vantage, Thermo Fischer Scientific). The HOat/HATU coupling system was then used to conjugate PF127 with the peptide. This approach involves the formation of a highly reactive ester with the help of 1-hydroxy-7-azabenzotriazole (HOat). The hydroxyl group of the PF127 was esterified by using this system. The reactive PF127 ester was coupled with the amine group of the peptide’s free amino acid. Purification and characterization steps similar to those for the pure peptide were followed.

The FITC-labeled peptide was prepared after Peptide–PF127 conjugation by using standard chemistry. The following peptide conjugates were synthesized:

STFTKSP (10:1 Peptide/PF127): purity >95%, MW: 766.81

STFTKSP (10:1 Peptide/PF127-FITC): purity >95%, MW: 766.81

The peptide was dissolved in acetonitrile/water (80:20) at 5 mg/ml and then diluted to 1 mg/ml in PBS. The HSCs cell suspension (1 × 10^4^ cells) was incubated with 50 μl of PF127–HSCBpep solution for 30–40 mins on ice. These cells are hence forth referred to as “PF127–HSCpep HSCs”. For CLSM, PF127–HSCBpep labeled with FITC was used. In brief, PF127–HSCBpep-FITC was added to the cell suspension and allowed to stand for 30–40 mins. After binding, the cells were fixed in 4% (w/v) PFA solution for 10 mins at 4 °C, washed twice with HBSS, and examined under a confocal microscope (Olympus Fluoview FV1000).

#### SEM

The shape and surface morphology of the PF127–CA HSCs and PF127–HSCBpep HSCs were discerned using SEM. A drop of the coated cells was placed on a glass cover slip that had been previously attached to a metallic stub (standard pin mount, aluminium, grooved edge) with a bi-adhesive carbon tape. The drop was air-dried and coated with gold particles to obtain a conducting surface. The sample was analysed using a Zeiss EVO-40 Scanning Electron Microscope operated in vacuum mode.

#### Cytokine permeability assay

A cytokine permeability assay was performed for the PF127–HSCBpep HSCs. For this protocol, cytokine GMCSF (16 kDa) was chosen. Initially, the cells (1 × 10^4^/ml) were treated with the respective cytokines for 30 mins and further incubated with 100% serum for 3hrs to degrade the layers and detect the presence of the cytokine that had crossed the wrap (coat) using anti-cytokine antibodies. The samples were examined using a flow cytometer (BD FACSAria III), and the mean fluorescence intensity was recorded. Each experiment was conducted in triplicates (n = 3), and the data were represented as mean ± SD.

### Zeta potential measurement

The zeta potentials of PF127-CA HSCs were measured to detect the changes in surface electrical charge characteristics. A cationic layer has a higher zeta potential in comparison with the control. On the other hand, an anionic layer has a lower zeta potential then the control. The respective zeta potentials after each coating were recorded in the suspension on a Zetasizer (MalvernZS90, UK) using the electrophoretic light scattering technique and a graph was plotted. Each experiment was conducted in triplicates (n = 3), and the data were represented as mean ± SD.

### Quantitative estimation of PF127 coated on PF127-CA and PF127-HSCBpep HSCs

The exact quantity of PF127 present on the cells after coating with PF127-CA and PF127-HSCBpep was estimated using the cobalt thiocyanate assay effective in detecting very low percentages of pluronics^29^. The cells were coated with PF127-CA and PF127-HSCBpep. The layers were degraded by incubation with 100% serum for 5 hrs. The amount of pluronics released in the serum was detected using the cobalt thiocyanate reagent (3 gm of cobalt nitrate and 20 gm of ammonium thiocyanate in 100 ml water). Briefly, PF127 test sample (serum containing pluronics), 100 µl cobalt thiocyanate reagent, 200 µl ethyl acetate, and 80 µl absolute ethanol were mixed well in a 2 ml microcentrifuge tube and then centrifuged for 5 mins at 10,000 rpm. After centrifugation, the upper two layers were aspirated using a 200 µl pipette. The pellet and the walls of the tube were washed with 200 µl of ethyl acetate to remove the excess reagent. The solution was again centrifuged at 10,000 rpm for 5 mins. The light blue supernatant obtained after the centrifugation was removed, and this step was repeated until it turned colorless. The pellet was dissolved in 1 ml of acetone, 200 µl of which was transferred to a 96-well plate. The absorbance was measured at 623 nm (Multi Skan Go, UV-Vis Spectrophotometer, Thermo Fisher Scientific), and the concentration of the PF127 was calculated using the standard curve (Supplementary Fig. 6). Each experiment was conducted in triplicates (n = 3), and the data were represented as mean ± SD.

### Treatment of PF127-CA HSCs with pMVs

The PF127-CA HSCs were incubated with the pMVs suspension (200 µl) for 2 hrs at 37 °C and also treated with human and mouse serum (200 µl). The cells were then centrifuged at 400 *g* for 5 mins. For the detection of bound pMVs, the cells were further incubated with anti-human and anti-mouse P-Selectin antibodies (CD62P-FITC; BD Biosciences 555523 and 553744, respectively) for 30 mins at 4 °C and viewed under a confocal microscope (Olympus Fluoview FV1000) after counterstaining with 4’, 6-diamidino-2-phenylindole (DAPI).

### Detecting the expression of CXCR4 in the pMVs-treated PF127-CA HSCs

The existence of the receptor/molecule CXCR4 on the HSCs and PF127-CA HSCs was subsequently evaluated through flow cytometry using an antigen-specific antibody. Briefly, the HSCs were encapsulated with PF127-CA. After washing, both HSCs and PF127-CA HSCs were treated with pMVs using the above-mentioned procedure. After incubation with pMVs, the unbound ones were removed by washing, and the cells were incubated with anti-human CXCR4 antibody (CD184-PE; BD Biosciences). After 1 hr, the cells were washed with HBSS and analyzed by flow cytometry (BD FACSAria III). Each experiment was conducted in triplicates (n = 3), and the data were represented as mean ± SD.

### TVM assay

All migration experiments were performed by using cell culture 12-well Transwell filter inserts (1.1 cm^2^, 3.0 µm pore size, Millicell-Merck Millipore, USA). The filters were first coated with fibronectin using 1 mg/ml stock solution (Sigma-Aldrich, USA) diluted to 1:20 in MilliQ water, and 250 µl of it was added to the apical compartment of the insert. After incubating for 2 hrs at 37 °C, the fibronectin solution was removed and the inserts were dried in the hood for 30 mins. Human bone marrow endothelial cells (hBMEC; Celprogen, USA) were cultured over the fibronectin coated cell culture inserts until an endothelial layer was established. Human bone marrow endothelial cell growth medium with serum (Celprogen, USA) was added to both the apical and basolateral compartment of the inserts in a 12-well plate. The cells at a density of 1 × 10^5^/ml were added to the medium and cultured inside a water-jacketed CO_2_ incubator (5% CO_2_, 95% humidity, and 37 °C temperature). The medium was changed once in 3 days. The confluent endothelial monolayers on the Transwell filters were washed thrice with the medium and placed in wells containing fresh medium. The PF127-CA HSCs treated with the pMVs suspension were seeded carefully on the insert without disturbing the endothelial layer present on them. After 8 hrs incubation (5% CO_2_, 95% humidity, and 37 °C temperature), the insert was carefully removed. At the end of the experiment, the transmigrated cells recovered from the basolateral compartment were counted manually, and the percentage migration was calculated. Each experiment was conducted in triplicates (n = 3), and the data were represented as mean ± SD.

### Animal experiments

Animal handling and experiments involving mice and rabbits were carried out after obtaining approval from the Institutional Animal Ethics Committee (IAEC), Institute of Nuclear Medicine and Allied Sciences (INMAS), Defence Research and Development Organization (DRDO), Delhi, India with Protocol no: INM/IAEC/2019/02; dated 28/02/2019 and Registration No: 8/GO/RBi/S/99/CPCSEA. The studies were conducted in athymic (*BALB/c*) nude mice (female, 6–8 weeks old, and weight 22 ± 2 gm) and New Zealand White rabbits (female, 4–5 months old and weight 3.5 ± 0.39 kg). All efforts were taken to minimize the number of animals and their suffering. Appropriate radiation doses as a part of the preconditioning measure were given to the mice before each experiment to further suppress their immune system for better results. The nude mice were housed in the INMAS Experimental Animal Facility (IEAF) at an ambient temperature of 30–32 °C with 45% humidity and supplied with food and water *ad libitum*. The New Zealand White rabbits were placed in cages with food and water available at all times and maintained under standard conditions with ambient temperature of 25 ± 2 °C and a regular 12 hr light/dark cycle. All experiments were performed according to the protocols approved by the Committee on the Ethics of Animal Experiments of INMAS, Delhi, India.

### *In vivo* bone marrow TVM studies

#### Infusion and detection of the transmigrated cells

Firstly, the HSCs were labeled with the PKH26 cell membrane dye (Sigma–Aldrich) as per the manufacturer’s protocol. Further, 1.6 × 10^4^ PKH26-labeled HSCs were encapsulated separately with PF127–CA and PF127–HSCBpep and intravenously injected into the nude mice irradiated with a conditioning radiation dose of 3.5 Gy or a lethal dose of 7.5 Gy using a ^60^Co-γ source irradiator (Bhabatron-II Teletherapy Unit, Panacea Medical Technologies, Bengaluru, India). After 24 hrs, the mice were sacrificed, the bone marrow was aspirated, and the cells present per unit volume were counted using a flow cytometer (MoFlo Cytomation, Beckman Coulter). Besides, assay was also performed to measure the ability of the other group of pluronics, PF68, and the long-circulation compound, PEO, to promote TVM, and the results were compared. The PKH26-labeled HSCs covered with the CA template were over-layered with PF127, PF68 and PEO and infused into the nude mice. After 24 hrs, the animals were sacrificed, and the bone marrow cells were analysed by flow cytometry. The experiments were conducted with six mice in each group (n = 6), and the data were represented as mean ± SD.

#### Histology

To evaluate the *in vivo* toxicity, the BALB/c/mice (male, 6–8 weeks old, and weight 24 ± 1.9 gm) were infused intravenously with PF127-CA and PF127-HSCBpep HSCs. Liver and spleen tissues were isolated by sacrificing the mice 1 week after infusion. The tissues were fixed in 4% formalin and embedded in paraffin. Sections of 5 µm thickness were cut and placed on glass slides, stained with haematoxylin and eosin, and the histopathological changes were observed under a microscope (BX60, Olympus).

### Bone marrow-specific TVM of the PF127 complex

We attempted to ascertain whether the TVM of the HSCs in the bone marrow is receptor-mediated, that is, does PF127 merely mediate the delivery or itself enters the bone marrow and carries the HSCs along with it. Initially, FITC-labeled PF127 was synthesized^11^, and the PKH26-labeled HSCs were encapsulated with the FITC-PF127-CA complex. Later, 1 × 10^4^ cells were injected intravenously into the athymic nude mice. The bone marrow suspensions were aspirated at time intervals of 0, 6, 10, 14, 16, 20 and 24 hrs and examined for the presence of cells containing the FITC-PF127 nanocomplex by using dual-channel flow cytometry (BD FACSAria III).

### Bone marrow histology studies

Furthermore, through histological studies, we tried to visualize the HSCs that were able to enter the bone marrow. Besides, we were also interested in checking whether the pMVs move into the bone marrow along with the PF127-CA HSCs. In this experiment, PF127-FITC was utilized, and the PF127-CA HSCs (pMVs treated and non-treated) were injected into nude mice that were previously exposed to conditioning radiation doses (3.5 Gy). After 6 and 8 hrs, the mice were sacrificed, the femur bones were excised, and sectioning was performed by employing the protocol as described earlier^48,49^ with slight modifications. Briefly, the femur bones from the hind legs were dissected and placed in 10% formaldehyde solution overnight. The bones were decalcified for 4–7 days using 14% ethylene diamine tetra acetic acid (EDTA) (pH 7.1). The decalcified femurs were then dehydrated with gradually increasing concentrations of ethanol from 50% to 100%, followed by washing with xylene. The femurs were embedded in paraffin, and longitudinal sections of 5 µm thickness were cut through the middle of the femur and placed on a glass slide. The bound pMVs were detected by treating the sections with PE-conjugated CD62P antibody. The slides were visualized under a fluorescence microscope (Zeiss) and checked for both FITC and PE fluorescence. The entire procedure was executed in the dark.

### TVM in the presence of CXCR4 antagonist AMD3100

To prove the involvement of CXCR4/SDF-1, the CXCR4 antagonist AMD3100 was used in both *invitro* transendothelial migration and in *invivo* homing experiments. For the former studies, BMEC layers were established over the 3 µm pore size fibronectin-coated insert as previously mentioned. The HSCs and PF127-CA HSCs were treated with pMVs suspension and subsequently with AMD3100 at a concentration of 2 µg/ml. Both HSCs and PF127-CA HSCs (AMD3100 treated and non-treated) were carefully seeded on the insert without disturbing the endothelial layer present on it. After incubating for 8 hrs (5% CO_2_, 95% humidity, and 37 °C temperature), the insert was carefully removed. At the end of the experiment, the transmigrated HSCs recovered from the basolateral compartment were counted manually and percentage migration was calculated. Each experiment was conducted in triplicates (n = 3), and the data were represented as mean ± SD.

The transendothelial migration experiment was followed by *in vivo* homing studies. The HSCs were first labeled with the PKH26 cell membrane dye (Sigma–Aldrich) according to the manufacturer’s protocol. Later, 1.6 × 10^4^ PKH26-labeled HSCs and PF127-CA HSCs were intravenously injected intraperitoneally into the non-AMD3100 treated and AMD3100 treated nude mice (400 µg) After 24 hrs, the mice were sacrificed, the bone marrow was aspirated, and the number of cells/unit volume of the aspirated bone marrow was counted using a flow cytometer (BD FACSAria III). The experiments were conducted with six mice in each group (n = 6), and the data were represented as mean ± SD.

### Regeneration studies

#### Detection of the regenerated human cells in mice bone marrow

Firstly, the nude mice were irradiated with a conditioning dose of 3.5 Gy or a lethal dose of 7.5 Gy (^60^Co). These animals were subsequently injected intravenously with 1 × 10^4^ PF127–CA and PF127–HSCBpep HSCs. An antibiotic solution of Sulfatrim (40 mg of trimethoprim and 200 mg of sulfamethoxazole per 5 ml) was added to the drinking water to avoid bacterial infections after irradiation. For proper regeneration of cells in the irradiated nude mice, the human recombinant cytokine combination TPO, SCF, IL-3, IL-6, and Flt-3, each amounting to 0.2 µg/kg was administered intraperitoneally thrice a week in phosphate-buffered saline (PBS) for the entire duration of the study, and the procedure began 24 hrs after infusion^50^. After 2 weeks, the mice were sacrificed, and the percentage of regenerated human cells in their bone marrow was examined using the mouse monoclonal human mitochondria-specific antibody (Abcam-ab92824) according to the manufacturer’s protocol with slight modifications. In brief, the cells were fixed with 4% formaldehyde for 10 mins and incubated with 1% BSA in 0.1% Tween-20 for 1 hr to block the non-specific protein–protein interactions and permeabilize the cells. These cells were then incubated with ab92824 (1:100 dilution) for 30 mins at 4 °C and later with the secondary antibody goat anti-mouse IgG; H&L; DyLight488 (ab96879, 1:500 dilution) and analysed by flow cytometry (MoFlo Cytomation, Beckman Coulter). The experiments were conducted with six mice in each group (n = 6), and the data were represented as mean ± SD.

#### Detection of human terminal lineage cells in mice peripheral blood

The flow cytometric detection of the human cells in mouse blood representing the terminal lineages stemming from the infused human cells was performed directly by using FITC-conjugated monoclonal antibodies to huCD45^+^, huCD41a^+^ and huCD15^+^ for leukocytes, platelets and neutrophils, respectively. Peripheral blood was obtained from the nude mice 2 and 4 weeks later through cardiac puncture, and 20 µl of antibody solution was added to 20 µl of EDTA-anticoagulated blood. After 30 mins of incubation at room temperature, these cells were fixed using 4% PFA. The red blood cells were lysed using RBC lysis buffer (8.26 gm NH_4_Cl, 1 gm KHCO_3_, and 0.037 gm EDTA in 1 litre of deionized water; autoclaved and stored at 4 °C). The cells were washed, diluted with 300 µl HBSS, and analysed using flow cytometry (MoFlo Cytomation, Beckman Coulter). The experiments were conducted with six mice in each group (n = 6), and the data were represented as mean ± SD.

### Survival studies

Female nude mice which were 6–8 weeks old and weighed 21 ± 2.6 gm were divided into groups of six, one for each time point used, to assess the survival and weight changes. These animals were first irradiated with a lethal radiation dose of 7.5 Gy (^60^Co) and then infused with 1 × 10^4^ HSCs, PF127-CA HSCs and PF127-HSCBpep HSCs. The mice were subsequently monitored on a daily basis, and their weights were recorded every third day. Survival was documented up to 60 days post-irradiation and expressed as a percentage. Weight loss or gain was directly expressed as weight (in gm) and noted up to 30 days. These experiments were conducted with six mice in each group (n = 6), and the data were represented as mean ± SD.

### Computation of SRC, LTC-ICs

The limiting dilution assay performed by Sutherland *et al*.^51^ and Bhatia *et al*.^31^ for the computational analysis of SRC and LTC-IC was employed. We carried out this procedure to estimate the numbers of these cells that could have transmigrated into the bone marrow. The major assumption in this model is that the successful transplantation of few cells of one regenerative cell type (i.e., SRC, etc.) is sufficient for a complete regenerative response. We then extended this computational ideology to our PF127 findings to establish the numbers of SRCs and LTC-ICs that existed within the pure HSCs populations infused and the HSCs when nanocomplexed. Their respective ratios were used.

### Rabbit bone marrow TVM studies

To address the validity of the results obtained in nude mice with a higher animal model, the delivery experiments using PF127 were performed on rabbits as they are phylogenetically similar to humans and their bone marrow has a sinusoidal ultrastructure as that of humans. For this purpose, 1.6 × 10^4^ PKH26-labeled HSCs and PF127-CA HSCs were infused into female New Zealand white rabbits which were 4–5 months old, weighed 3.5 ± 0.39 kg, and were immunosuppressed with cyclophosphamide (60–80 mg/kg bw). After 24 hrs, their bone marrow was aspirated from the femur bone using a 10 ml syringe equipped with Rosenthal bone marrow aspiration needle and observed by flow cytometry (BD FACSAria III). The experiments were conducted in three rabbits for each group (n = 3), and the data were represented as mean ± SD.

### Statistical analysis

All the results have been presented as mean ± SD. All statistical analyses were performed on Microsoft Excel; one way Analysis of Variance (ANOVA) was used followed by Boneferroni’s multiple-comparison post-hoc tests. Differences with a p-value < 0.05 were considered statistically significant.

## Supplementary information

Supplementary information.
