# Supplementary material for: Pluronic-F127/Platelet Microvesicles nanocomplex delivers stem cells in high doses to the bone marrow and confers post-irradiation survival
Source: Sci Rep. 2020 Jan 13;10:156. doi: 10.1038/s41598-019-57057-8 (PMC6957521; doi:10.1038/s41598-019-57057-8)
Supplement: Supplementary file 1 — Supplementary information. [file 41598_2019_57057_MOESM1_ESM.pdf]

ARTICLE TYPE:

Original Research Article- **supplementary information**

TITLE:

Pluronic-F127/Platelet Microvesicles nanocomplex delivers stem cells in high doses to the bone marrow and confers post-irradiation survival

INFORMATION:

Authors List: Vikas Chander, Gurudutta Gangenahalli*

First Author: Vikas Chander

Second Author: Gurudutta Gangenahalli*

CORRESPONDING AUTHOR (*)

Dr.Gurudutta Gangenahalli, FNA Biol. Sci., FRAMI, FICI, FRACI, Fellow of the Royal Society of Medicine (UK), FRSB (UK), FRSC (UK) & Scientist 'G', Head of the Division of Stem Cell Gene Therapy Research, Institute of Nuclear Medicine & Allied Sciences (INMAS), Delhi-110054, Phone: 91-11-23905144, Fax: 91-11-23919509, Email:gugdutta@rediffmail.com

AFFILIATE INSTITUTIONS:

Division of Stem Cell and Gene Therapy Research, Institute of Nuclear Medicine and Allied Sciences, Defence Research and Development Organization, Delhi-110054

Pluronic-F127/Platelet Microvesicles nanocomplex delivers stem cells in high doses to the bone marrow and confers post-irradiation survival

Vikas Chander[1], *Gurudutta Gangenahalli*[2]*

[1,2] Division of Stem Cell & Gene Therapy Research, Institute of Nuclear Medicine & Allied Sciences, Delhi-110054, India

***Correspondence**:

Dr.Gurudutta Gangenahalli, FNA Biol. Sci., FRAMI, FICI, FRACI, Fellow of the Royal Society of Medicine (UK), FRSB (UK), FRSC (UK) & Scientist 'G', Head of the Division of Stem Cell Gene Therapy Research, Institute of Nuclear Medicine & Allied Sciences (INMAS), Delhi-110054, Phone: 91-11-23905144, Fax: 91-11-23919509, Email:gugdutta@rediffmail.com

SUPPORTING INFORMATION:

Materials:

Chemicals and Recombinant Proteins			
**S.No.**	**Name**	**Source**	**Identifier**
1.	Chitosan	Sigma-Aldrich	Cat#C3646
2.	Alginate	Sigma-Aldrich	Cat#A2033
3.	Pluronic F127/Poloxamer407 (P407)	Sigma-Aldrich	Cat#16758
4.	Pluronic F68/Poloxamer 188 (P188)	Sigma-Aldrich	Cat#15759
5.	PEG	Sigma-Aldrich	Cat#1546569
6.	Poly-L- Lysine (PLL)	Sigma-Aldrich	Cat#P8920
7.	Thrombin	MP Biomedicals	Cat#101141
8.	Flt-3 Ligand Protein	Merck Millipore	Cat#GF038
9.	Stem Cell Factor Protein	Merck Millipore	Cat#GF021
10.	Granulocyte-Macrophage Colony-Stimulating Factor Protein	Merck Millipore	Cat#GF004
11.	Interleukin-3 Protein	Merck Millipore	Cat#IL003
12.	Fluorescein isothiocyanate isomer I	Sigma-Aldrich	Cat#F7250
13.	Rhodamine-β-isothiocyanate	HiMedia	Cat#RM2426
14.	AMD3100 octahydrochloride hydrate	Sigma-Aldrich	Cat#A5602
15.	SDF-1 protein	Abcam	Cat#ab85487
16.	Sodium thiocyanate	Sigma-Aldrich	Cat#251410
17.	Cobalt nitrate	Sigma-Aldrich	Cat#239267
18.	Ammonium thiocyanate	Sigma-Aldrich	Cat#221988

Antibodies			
**S.No.**	**Name**	**Source**	**Identifier**
1.	Anti -Human CD62P-FITC	BD Biosciences	Cat#555523
2.	Anti-Mouse CD62P-FITC	BD Biosciences	Cat# 553744
3.	Anti-Human FLT3 Ligand	Abcam	Cat#ab52648
4.	Anti-Human SCF	Abcam	Cat#ab64677
5.	Anti-human-IL-3	Abcam	Cat#ab9620
6.	Anti-Human GMCSF PE	Biolegend	Cat#502306
7.	Goat Anti-Rabbit IgG H&L FITC	Abcam	Cat#ab6717

8	Anti-Human CD184-PE	BD Biosciences	Cat#555974
9.	Anti-Human Mitochondria antibody	Abcam	Cat#ab92824
10.	Anti-Mouse IgG H&L DyLight 488	Abcam	Cat#ab96879
11.	Anti-Human CD45-FITC	BD Biosciences	Cat#555482
12.	Anti-Human CD15-FITC	BD Biosciences	Cat#555401
13.	Anti-Human CD11a FITC	BD Biosciences	Cat#555379
14.	Anti-Human SDF-1	Abcam	Cat#ab155090

**Cell culture**			
**S.No.**	**Name**	**Source**	**Identifier**
1.	Iscove Modified Dulbecco's Medium	Sigma-Aldrich	Cat#I7633
2.	RPMI-1640	Sigma-Aldrich	Cat#R6504
3.	K-562 Cells line	ATCC	CCL-243
4.	Human Bone Marrow $CD34^{+}$ cells	Lonza	Cat#2M-101
5.	Human Bone Marrow Endothelial cells	Celprogen	Cat#36095-24
6.	Human Bone Marrow Endothelial Medium	Celprogen	Cat#M36095-24S
7.	Antibiotic solution	Himedia	Cat#A002

**Animals**	
1.	BALB/c mice
2.	Nude mice (*nu/nu*)
3.	New Zealand White rabbits

**Other**			
**S.No.**	**Name**	**Source**	**Identifier**
1.	LIVE/DEAD Viability/Cytotoxicity Kit	Invitrogen	Cat#L3224
2.	Fetal Bovine Serum	Gibco	**Cat#10270**
3.	PrestoBlue cell viability reagent	Invitrogen	Cat#A1321
4.	MTT	Sigma-Aldrich	Cat#M2128
5.	Fibronectin	Sigma-Aldrich	Cat#F1141
6.	PKH26 Cell Membrane labeling kit (Red)	Sigma-Aldrich	Cat#PKH26GL

SUPPLEMENTARY RESULTS:

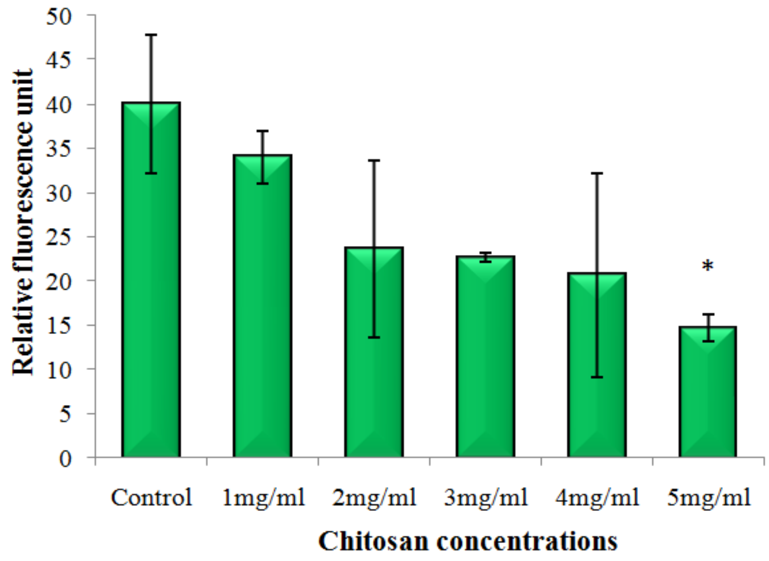

(A)

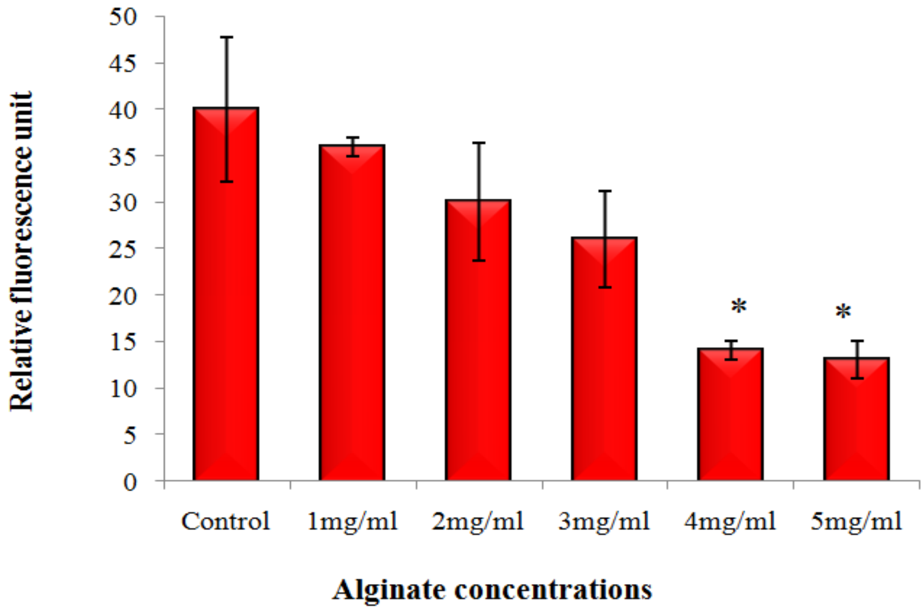

(B)

Supplementary figure.1 Bar graphs representing the results of the viability assay conducted using PrestoBlue cell viability reagent. PrestoBlue cell viability microplate assay was performed which uses the reducing power of the cell to quantitatively measure the proliferation potential of cells and thereby ascertaining the cell viability. Initially, the minimal concentration of **(A)** chitosan & **(B)** alginate non-toxic to cells was calculated using a PrestoBlue assay. (*n*=3, results as mean±SD, *=$P\leq0.05$, #=$P\leq0.01$).

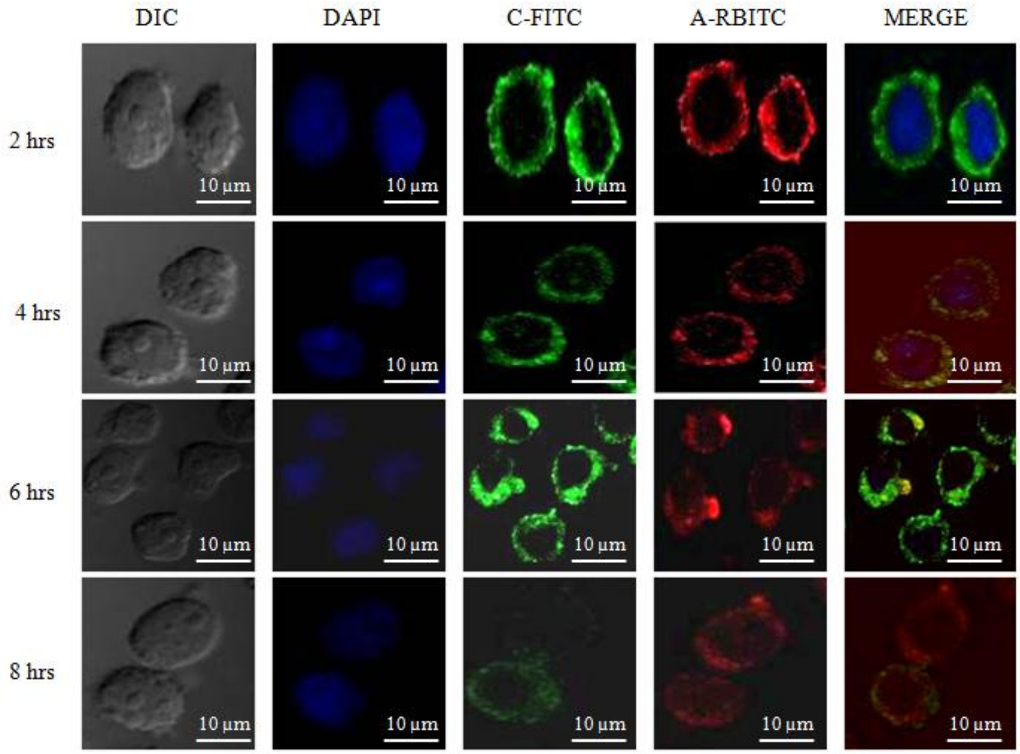

Supplementary figure.2 CLSM: Template stability studies- images of cells incubated in 100% serum at different time intervals. CA templated cells were incubated in 100% serum for 2, 4, 6 and 8 hrs to access the degradation. Cells started to lose their template after six hours, as observed under a confocal microscope. The fluorescence diminished upon further incubation, suggesting the presence of coating for up to eight hours. Scale: 10 µm. DAPI: 4′, 6-diamidino-2-phenylindole, UV Filter, Excitation/Emmision: 358/461, FITC: Fluorescein isothiocyanate, Blue filter, Excitation/Emission: 490/525, RBITC: Rhodamine-β-isothiocyanate, Green Filter, Excitation/Emission: 543/569.

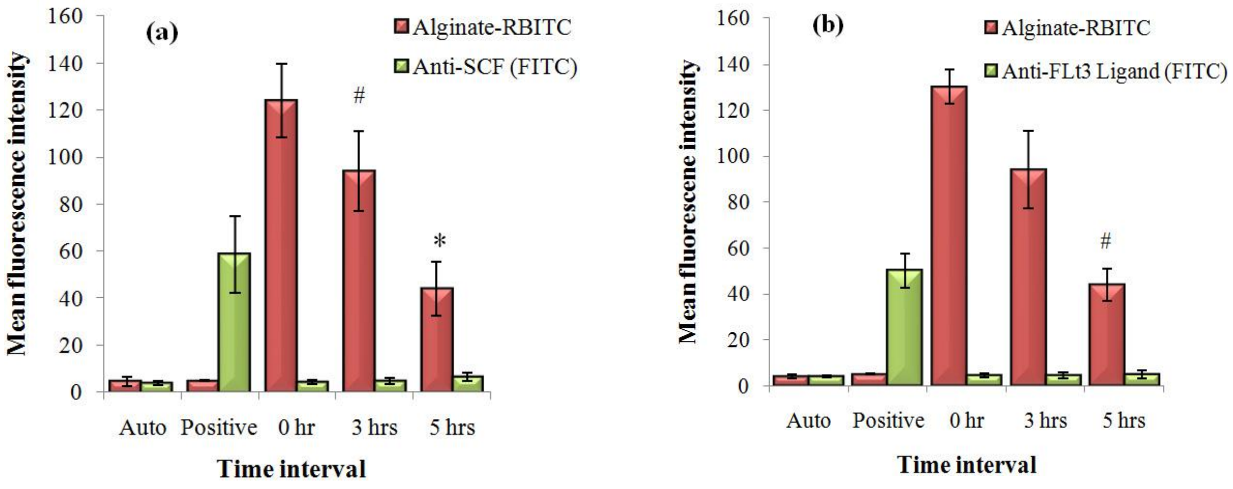

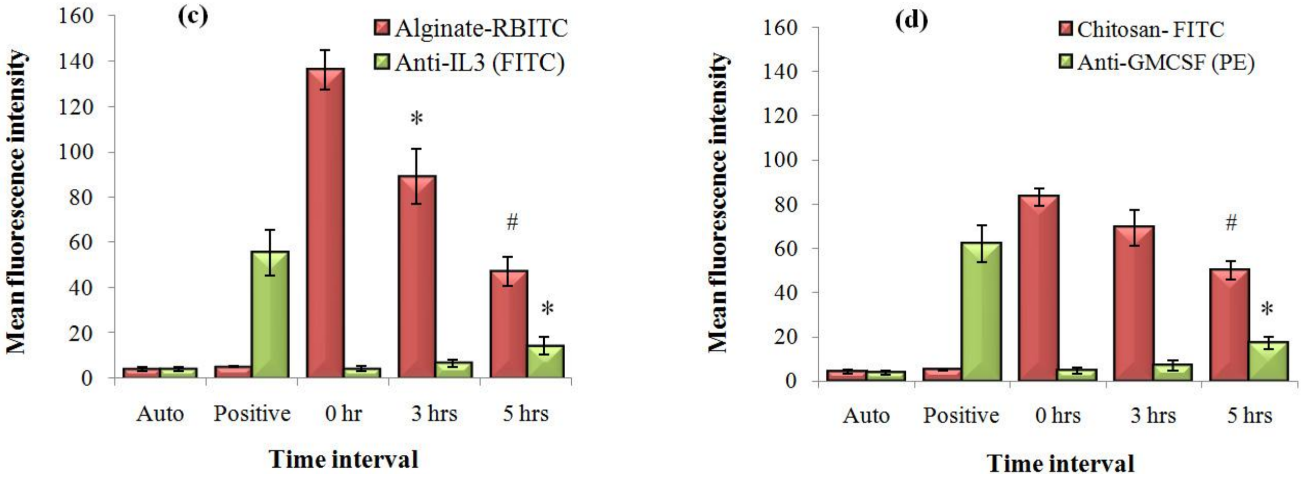

Supplementary figure.3 Flow Cytometry: **Cytokine permeability studies-** Four cytokines of different molecular weights were chosen for the assay: SCF, IL-3, Flt-3 ligand and GMCSF. The template was found to be impermeable to Flt-3 ligand (17.6 kDa) and SCF (18.6 kDa) but semipermeable to IL-3 (15 kDa) and GMCSF (16 kDa)**.** Bar graphs representing the effectiveness of coating material in avoiding contact with the stimulatory cytokines:(a) FLT-3 ligand, (b) SCF, (c) IL-3 & (d) GMSCF. All were detected inside cells using antibodies specific to these cytokines after degradation of the template (n=3, results as mean±SD,*=$P \leq 0.05$, #=$P \leq 0.01$).

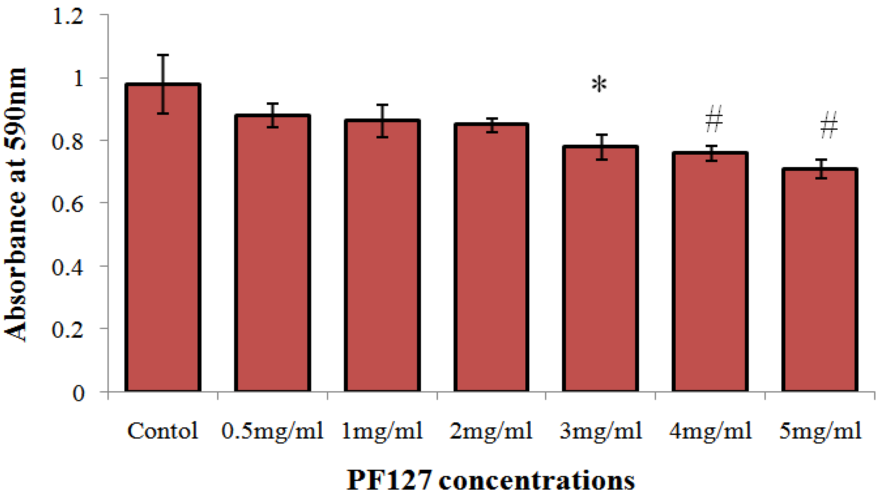

(a)

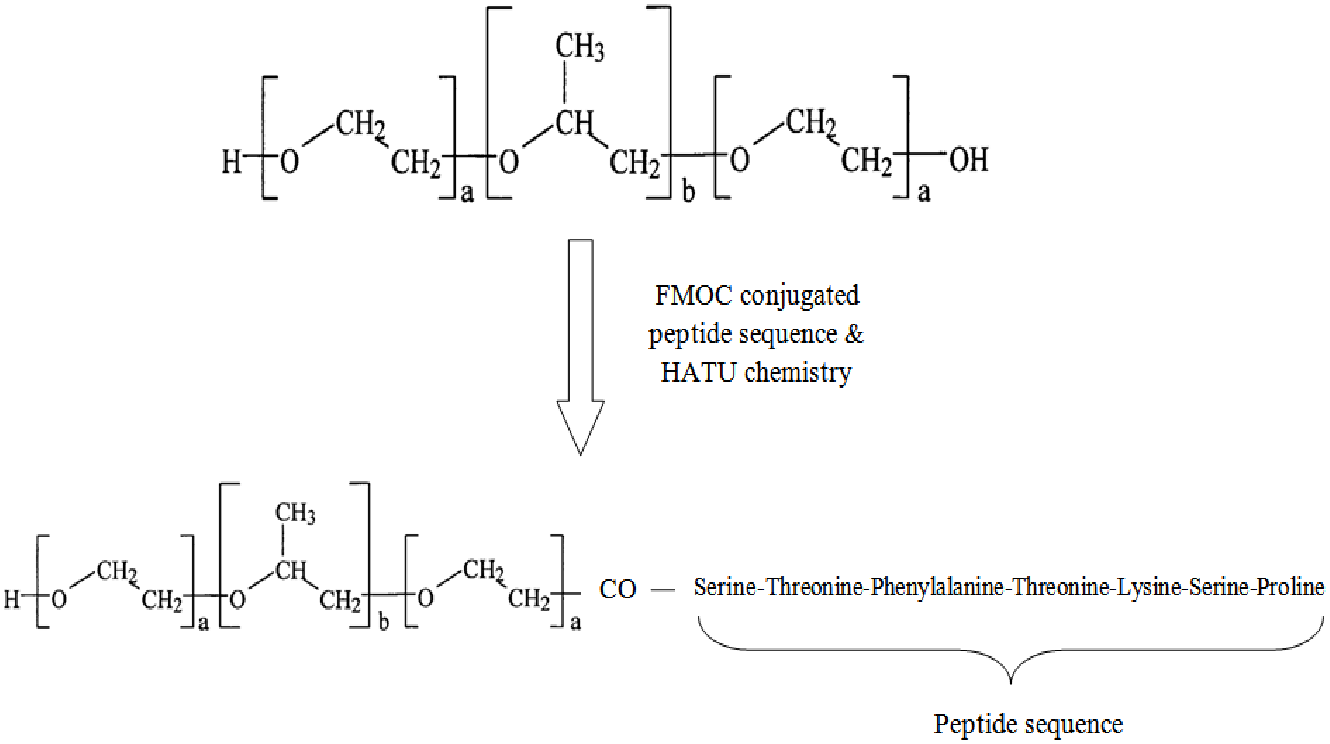

(b)

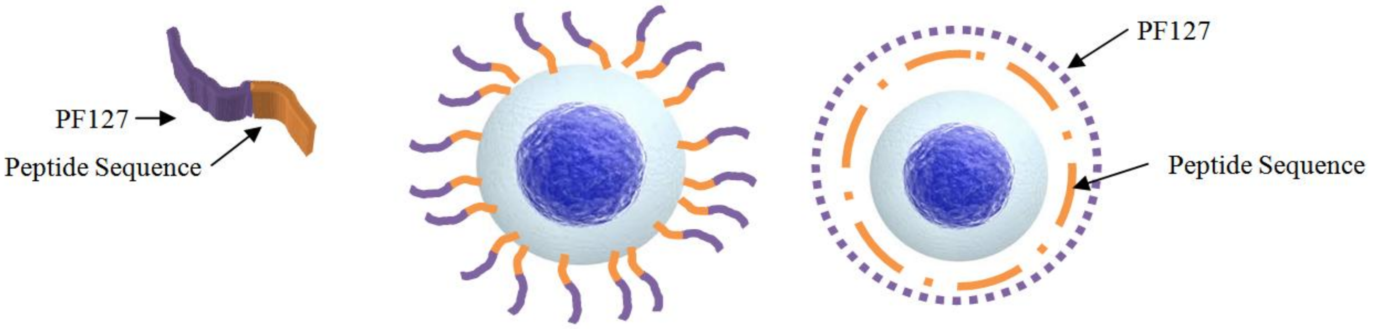

(c)

Supplementary figure.4 (a) Bar graph representing the results of MTT toxicity assay for determining the concentration of PF127 to be used (n=3, results as mean±SD,*=P ≤0.05, #=P ≤0.01). **(b)** Presumed structure after conjugation of PF127 with HSC binding peptide (HSCBpep) and **(c)** Binding of PF127-HSCBpep with the cells.

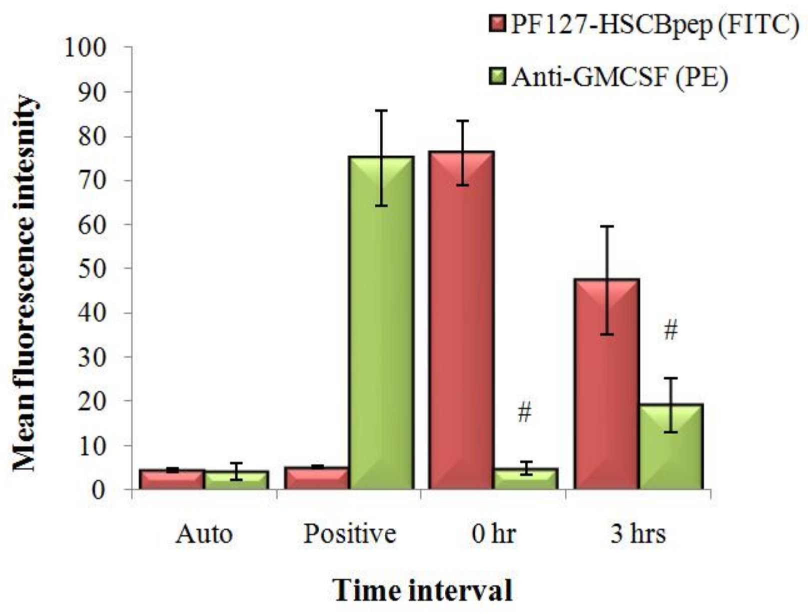

Supplementary figure.5 Flow Cytometry: Cytokine permeability studies- Bar graphs representing the effectiveness of PF127-HSCBpep in avoiding contact with GMCSF using antibodies specific to GMCSF after degradation of the coat (n=3, results as mean±SD,*=P ≤0.05, #=P ≤0.01).

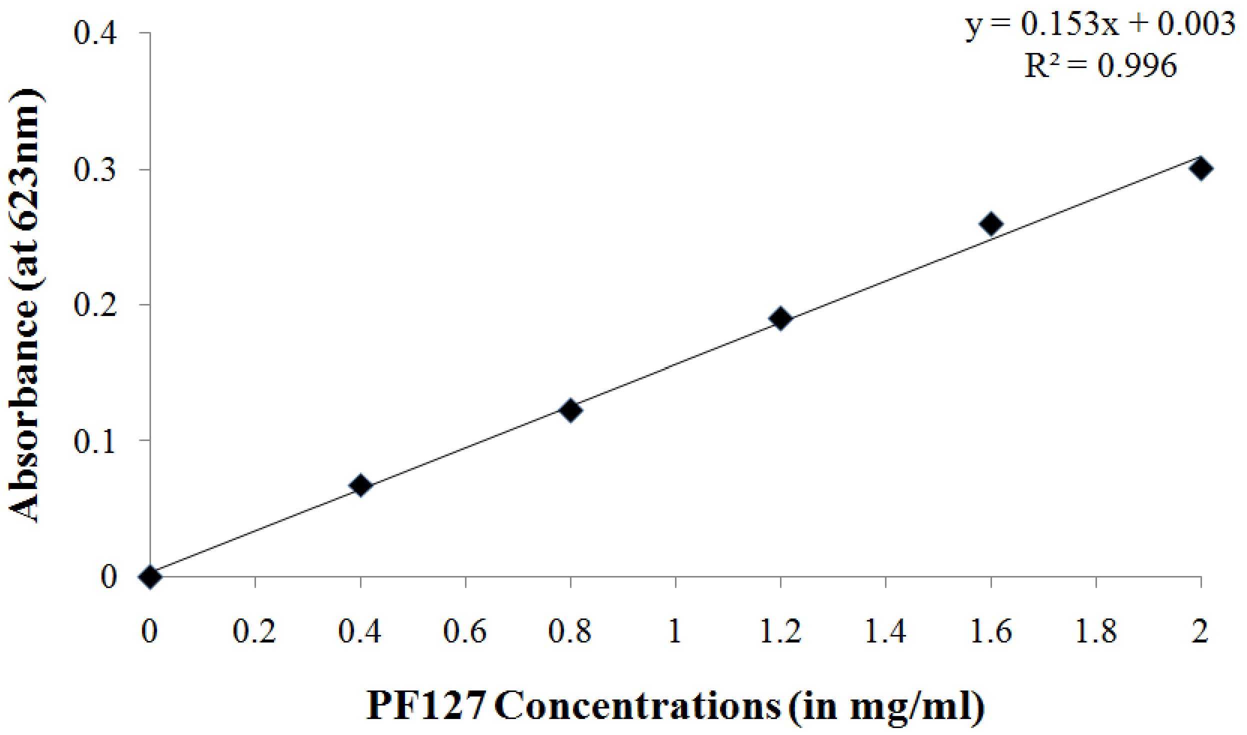

Supplementary figure.6 Standard curve/plot of PF127 using cobalt thiocyanate method

DEXTRAN IRON OXIDE NANOPARTICLES STUDIES:

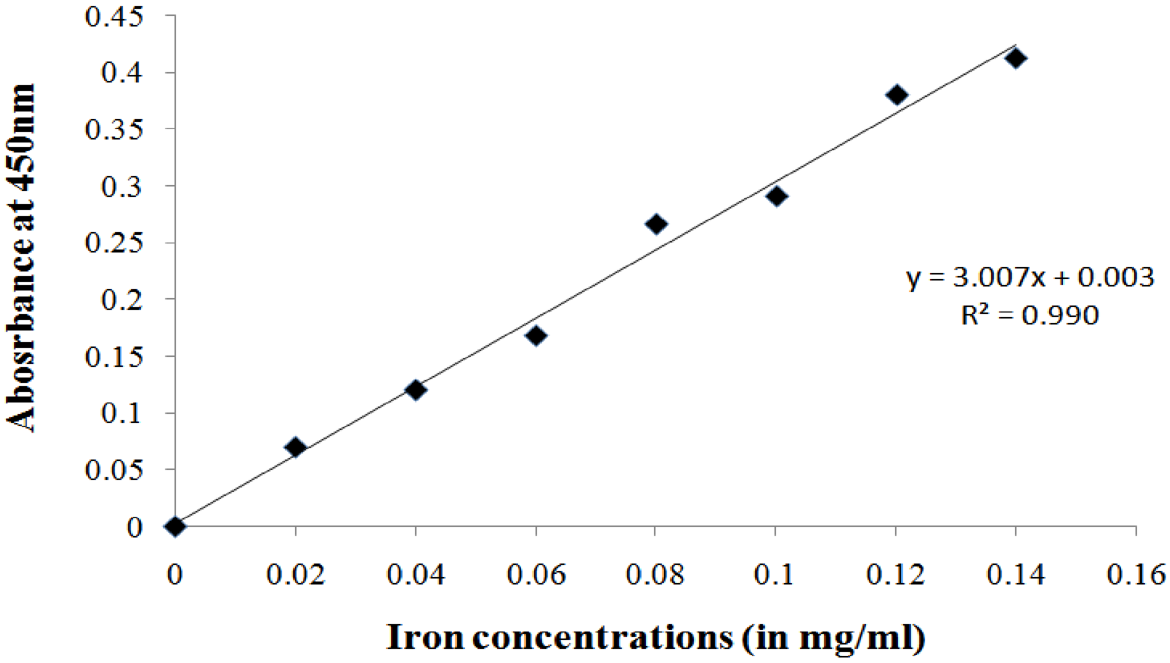

Supplementary figure.7.1 Standard curve/plot of iron by sodium thiocyanate method

The IOP's were synthesized by modified alkaline co-precipitation method (Hong *et al*, 2008; Hong *et al*, 2009) with slight modifications. For the preparation of IOP first 100mg $FeCl_2$ was weighed and dissolved in 5ml 2N HCl. Similarly, 175mg $FeCl_3$ was weighed and dissolved in 5ml 2N HCl. Both the solutions were sonicated for 15 mins using a probe sonicator (Biologics 3000, Ultrasonic Homogenizer). Solutions were then mixed by stirring for 1 hour; the solution was precipitated by adding 0.5N ammonium hydroxide until the black precipitate is formed followed by the addition of 4% Sodium Hypochlorite. The solution was centrifuged at 8000 RPM for 30 mins. A black precipitate obtained was dissolved in 10mg/ml solution of dextran (10ml) and stirred for 30 mins. The solution was dialyzed against deionized water for 24 hours with a change of water after every 8 hrs, the solution was again centrifuged at 8000rpm for 30 mins and the pellet was stored. Characterization of DexIOP was done by Dynamic light scattering (Malvern Zetasizer ZS90) and Transmission Electron Microscopy (TEM, JEOL 2100F). The concentration of Iron in DexIOP was determined by atomic absorption spectroscopy (AAS, GBC 932 scientific equipments).

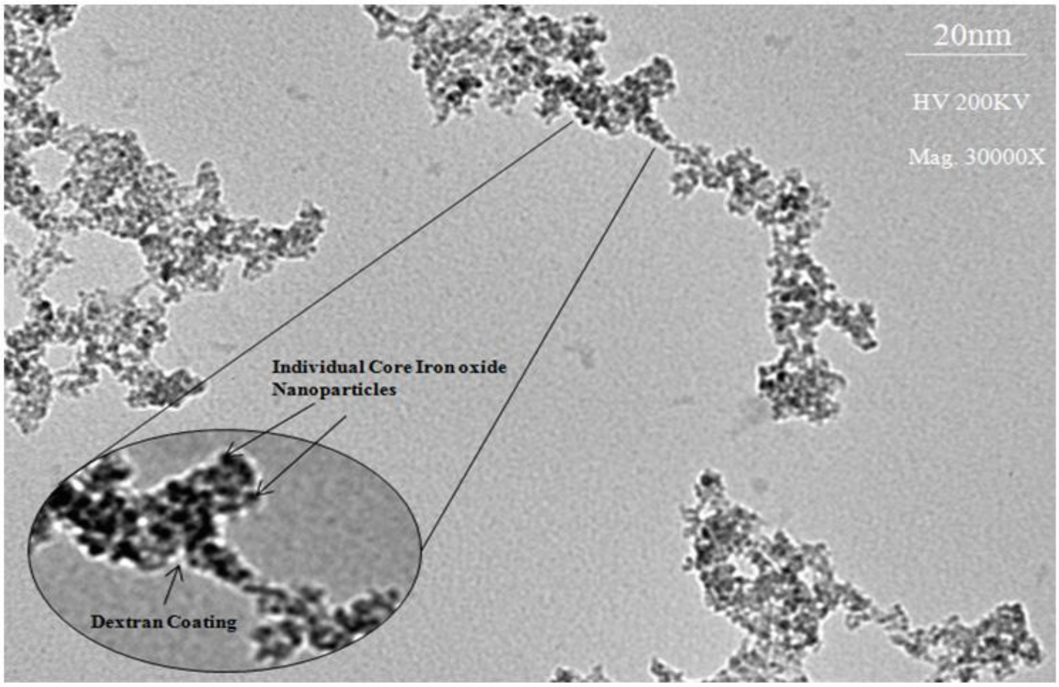

Supplementary figure.7.2 TEM: images of dextran iron oxide nanoparticles and their characterization

Concentrations of chemical compounds	
Fe^{2+}	100 mg
Fe^{3+}	175 mg
Dextran (10kDa)	100 mg

DLS Characterization	
Size (diameter)	164.9 nm
PDI	0.26
Zeta potential	21.2 mV
Size by TEM	43.nm

TVM studies on DexIOP labeled K-562 cells:

Cells were initially labeled with iron oxide nanoparticles and infused into nude mice. Briefly, iron oxide particles (100µg/ml) and Poly-L-Lysine (4µg/ml) were mixed and incubated for 15 mins for the formation of IOP-PLL complex. The complex was then added to the cells and

incubated for 30 mins initially in serum-free media, and the serum was subsequently added to the media to make the final conc. of serum to about 10%. Incubation was further carried out for 6-7 hrs. The presence of iron oxide inside cells after labeling was detected by Prussian blue staining (Arbab *et al*, 2004; Hu *et al*, 2009). Briefly, the labeled cells were washed with PBS and fixed in 4% paraformaldehyde for 10 mins. Cells were again washed with PBS and incubated with Prussian blue stain (4% potassium ferrocyanide in 3.7% hydrochloric acid) for 30 mins, rewashed with PBS and counterstained with nuclear fast red for 5 mins. The cells were observed under a bright-field microscope to determine intracellular iron oxide distribution.

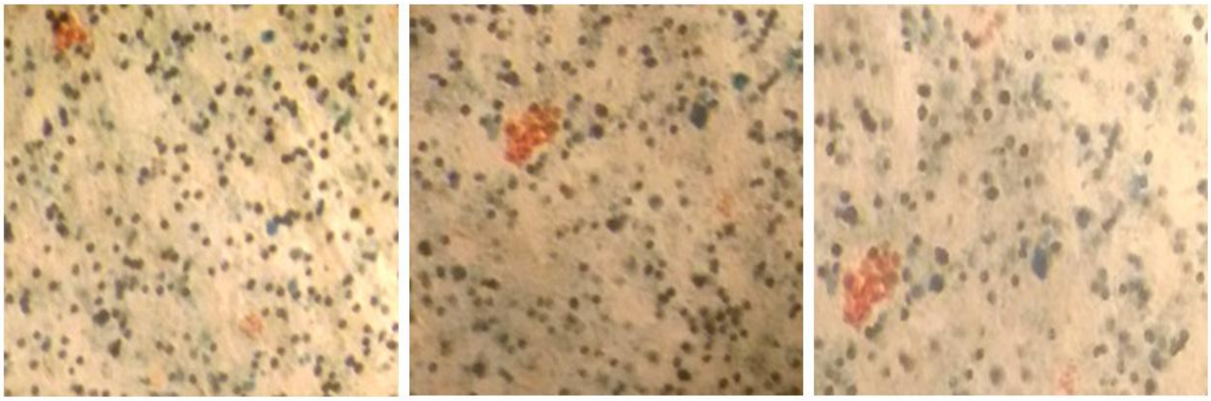

Supplementary figure 7.3 Prussian blue staining of iron oxide-labelled cells (objective: 10X)

IOP labeled cells were encapsulated with PF127-CA and were injected through tail vein (Intravenous-IV) into nude mice after 24 hrs post irradiation (6 Gy). After 24 hrs post infusion, mice were sacrificed and bone marrow (from femurs and tibias) and other organs lungs, liver, spleen, and tissue over the long bone are isolated. The tissues are ruptured with collagenase and digested using 6N HCl, and iron content in the supernatant was estimated by using sodium thiocyanate assay:

100µl of the supernatant was taken in 96 well plate to which 100µl reagent (0.1M Sodium thiocyanate) was added. Presence of iron gives red color which becomes intense with an increase in content. Readings were taken at 450 nm and content in micrograms was calculated through extrapolation with the standard.

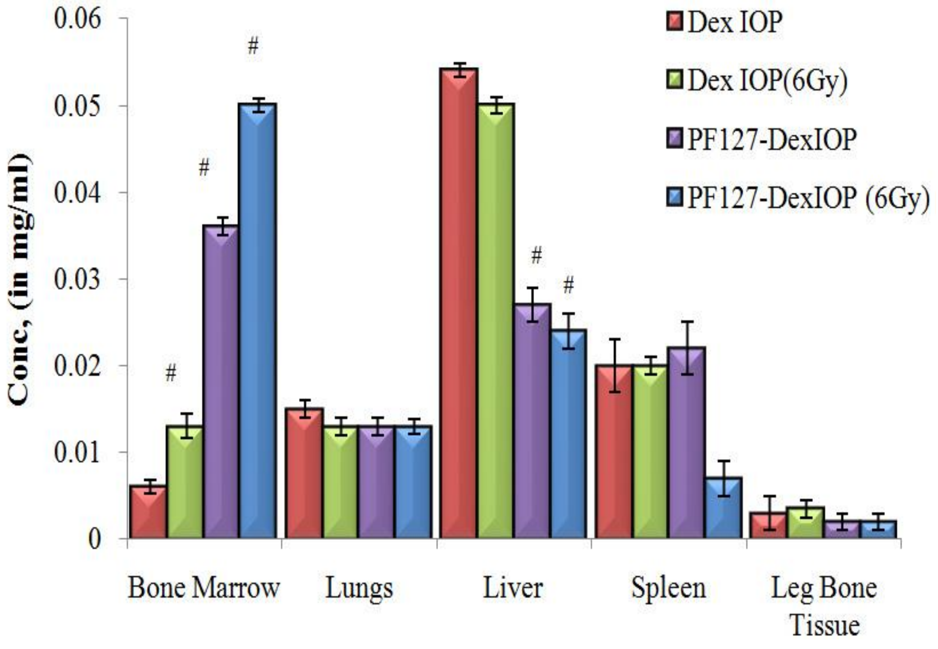

Supplementary figure.7.4 Iron content distribution in the bone marrow of nude mice, Bar graphs depicting the distribution of iron content in the bone marrow and other organs 24 hours after the infusion of iron oxide-labeled K-562 cells into nude mice. (**Iron Content: 101µg/1×10^{6} cells, estimated similarily by sodium thiocyanate assay after lysis of labeled cells)** (*n*=6, results as mean±SD,*=*P* ≤0.05, #=*P* ≤0.01).

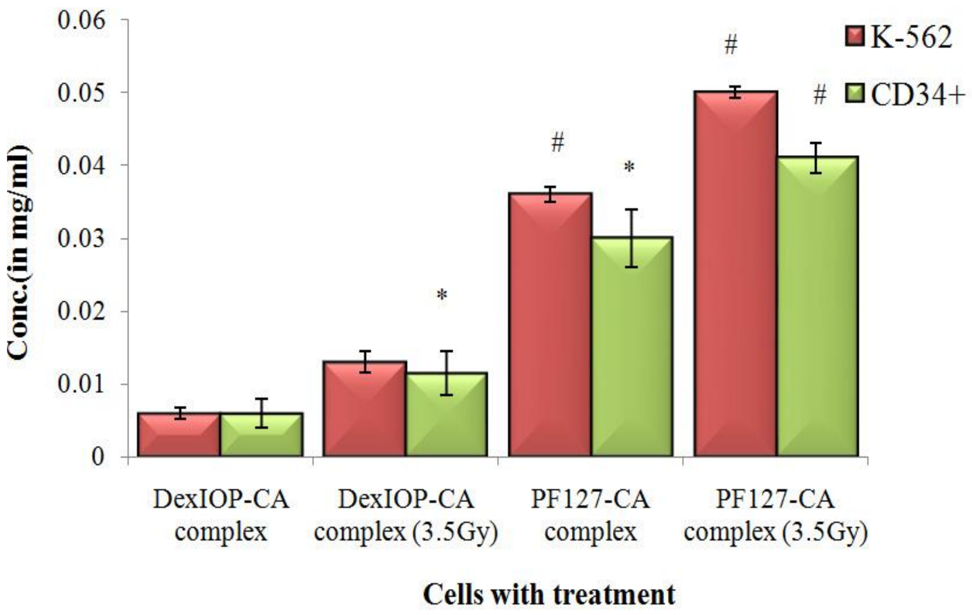

Supplementary figure.7.5 Comparative study on K-562 and huCD34^{+} cells, Bar graph comparing iron content in bone marrow after 24 hours when two different iron-oxide-labeled cells (K-562 and huCD34^{+}cells) were infused into nude mice after radiation conditioning (*n*=6, results as mean±SD,*=*P* ≤0.05, #=*P* ≤0.01).

Table. 1 The change in the percentage of different human cell types found in the peripheral blood of nude mice after two and four weeks. **C1** signifies the percentage comparison after two and four weeks between PF127–CA HSCs and non-wrapped HSCs, respectively, which clearly reflects the effectiveness of PF127 in speeding recovery and decreasing the recovery time. Similarly, **C2** shows the potency of PF127-HSCBpep HSCs in decreasing the recovery time compared with the data obtained from non-wrapped HSCs after four weeks of infusion. **C3** and **C4** compare the percentages of different lineages observed in the peripheral blood four weeks after infusion of PF127–CA HSCs and PF127-HSCBpep HSCs, respectively, with the results obtained by using non-encapsulated HSCs.

S.No	Cells infused	Percentage (%) regenerated human terminal lineage cells detected in nude mice peripheral blood after specific time interval							
		2 weeks				4 weeks			
		Leuk.	Plat.	Neut.		Leuk.	Plat.	Neut.	
1	HSCs	1.21±0.38	1.05±0.24	1.03±0.23		3.03±0.95	2.29±1.02	1.51±0.34	
2	PF127-CA HSCs (3.5Gy)	13.1±4.15	11.4±2.13	10.4±2.5	C1	32±5.5	30±3.05	25.3±5.1	C3
3	PF127-CA HSCs (7.5 Gy)	10.93±2.5	11.13±2.7	7.4±2.7	C2	23.1±3.2	19.64±3	12.25±3.8	C4
4	PF127-HSCBpep HSCs (3.5Gy)	4.7±2.2	4.9±1.8	9±3.1		29.3±4.5	20±5.2	23.8±4.7	
5	PF127-HSCBpep HSCs (7.5Gy)	9.3±2.1	7.1±1.9	4.3±1.5		26.13±5.8	19.6±5.13	9.6±3.2	

Supplementary data: Platelet microvesicles count:

pMVs were isolated as per the protocol mentioned by Janowska-Wieczorek *et al* and Forlow *et al*. For counting of pMVs, procedure mentioned by Nielsen *et al*, Springer *et al* and Robert *et al* was followed with few modifications; first isolated pMVs were labeled with CD62P-FITC. After calibration of the instrument with fluorescence beads (0.2-1µm, Thermo Fisher), samples were analyzed by flow cytometry (BD FACS aria 3). **Fig.8** shows one of the dot plot obtained after running the samples, which highlights the pMVs (in red) labeled with CD62P-FITC. Three readings were taken, which provided us with an average of 9048±170 pMVs in 50µl suspension which corresponded to 181±4.4 pMVs/µl.

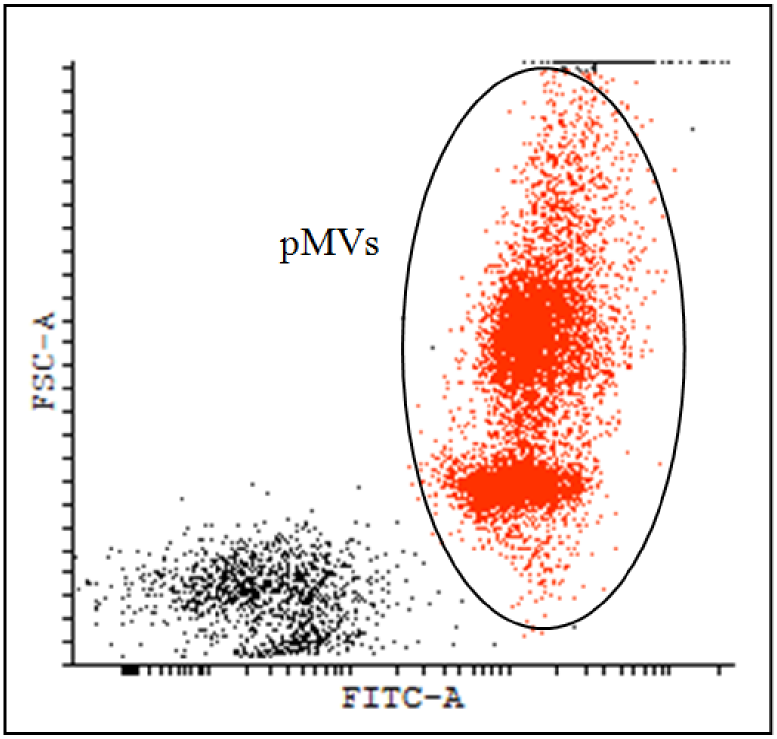

Supplementary figure.8 Flow Cytometry: Dot plot respresenting the results of pMVs count

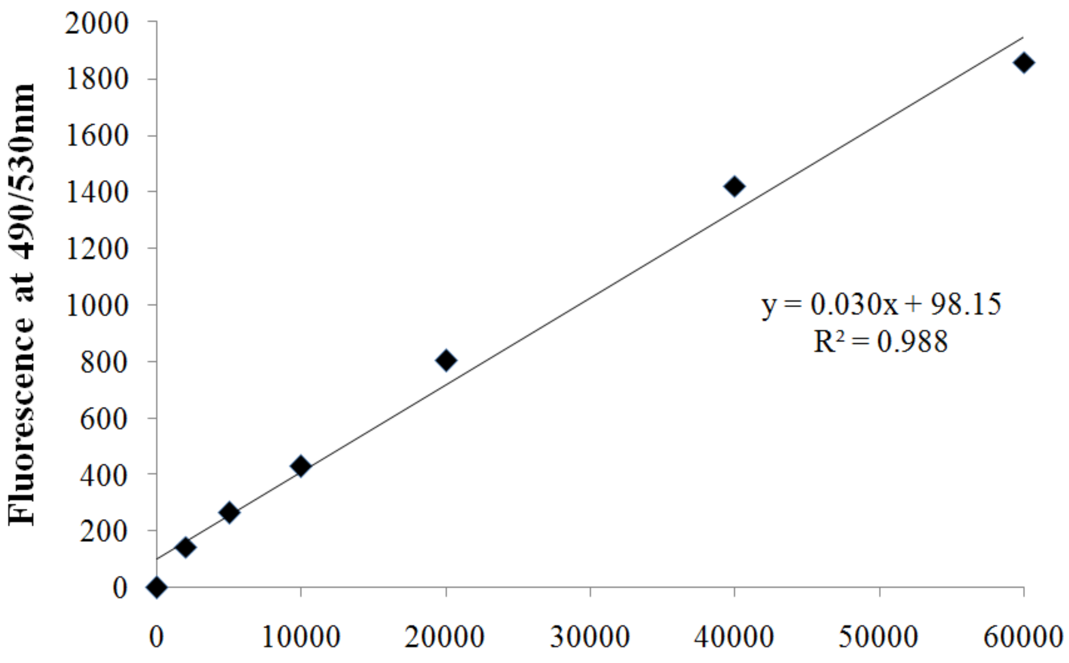

Supplementary figure.9 Standard curve obtained after plotting relative fluorescene unit against different number of pMVs. pMVs were initially labelled with CD62P-FITC for detection at excitation/emission: 490/530nm.

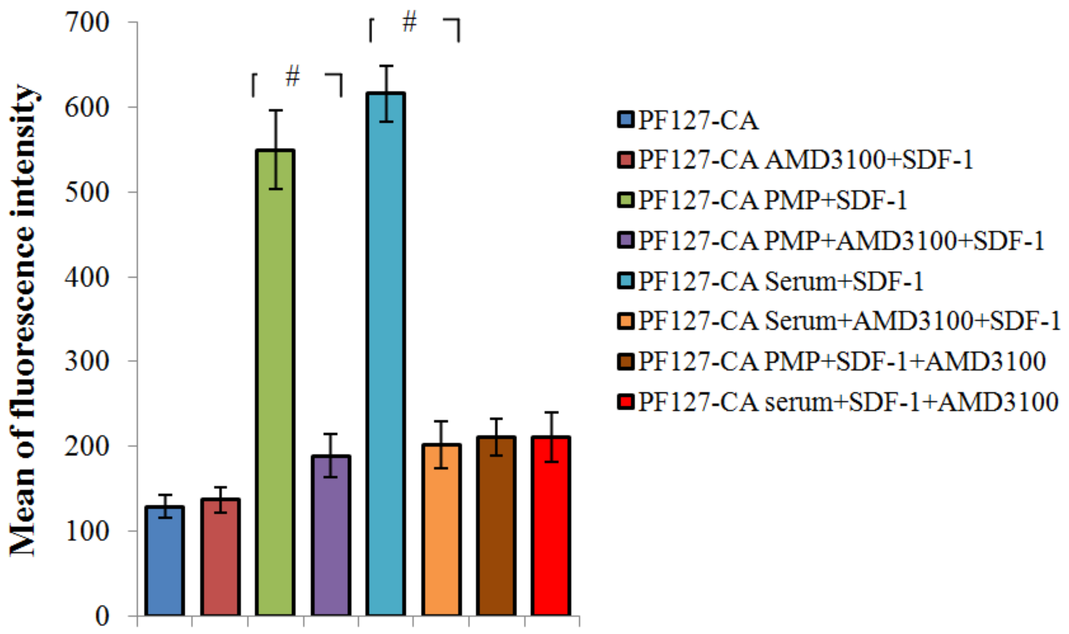

Supplementary figure.10 Flow cytometry: Bar graph representing the effect of AMD3100 in inhibiting the binding of SDF-1 to its receptor CXCR4. SDF-1 binding was checked by antibody to SDF-1 (#=$p \leq 0.01$)

REFERENCES:

1. Hong, R.Y, *et al*. Synthesis, characterization and MRI application of dextran-coated Fe3O4 magnetic nanoparticles. Biochemical Engineering Journal. **42**, 290-300 (2008).

2. Hong, R.Y., Li, J.H., Qu, J.M., Chen, L.L. & Li HZ. Preparation and characterization of magnetite/dextran nanocomposite used as a precursor of magnetic fluid. Chemical Engineering Journal. **150**, 572-580 (2009).

3. Mishra, S.K., Khushu, S. & Gangenahalli G. Potential stem cell labeling ability of poly-L-lysine complexed to ultra small iron oxide contrast agent: An optimization and relaxometry study. Exp Cell Res. **339**, 427-436 (2015).

4. Arbab, A.S. *et al*. In vivo trafficking and targeted delivery of magnetically labeled stem cells. Hum Gene Ther. **15**, 351-360 (2004).

5. Hu, S.L. *et al*. In vitro labeling of human umbilical cord mesenchymal stem cells with superparamagnetic iron oxide nanoparticles. J Cell Biochem. **108**, 529-535 (2009).

6. Nielsen, M.H., Beck-Nielsen, H., Andersen, M.N. & Handberg, A. A flow cytometric method for characterization of circulating cell-derived microparticles in plasma. J Extracell Vesicles. **3**, 1-12 (2014).

7. Springer, N.L., Smith, E., Brooks, M.B. & Stokol, T. Flow cytometric detection of circulating platelet-derived microparticles in healthy adult horses. Am J Vet Res. **75**, 879-885 (2014).

8. Robert, S. *et al*. Standardization of platelet-derived microparticle counting using calibrated beads and a Cytomics FC500 routine flow cytometer: a first step towards multicenter studies? J Thromb Haemost. 7, 190-197(2009).
